# Genetic Mechanism That Defines the Characteristic Neurogenesis Patterns in the Neural Plate Using *Hes/her* Genes During Early Vertebrate Development

**DOI:** 10.1002/dvg.70015

**Published:** 2025-06-02

**Authors:** Takero Ohyanagi, Hiroki Tokizaki, Takehisa Sato, Momo Tsuruoka, Kyo Yamasu

**Affiliations:** ^1^ Division of Life Science Graduate School of Science and Engineering, Saitama University Saitama Japan

**Keywords:** CRISPR/Cas9 method, *Hes/her*, neural progenitor cells, Notch signaling, primary neurogenesis, proneural cluster domain, zebrafish

## Abstract

In the early zebrafish neural plate, proneural cluster domains are defined by surrounding neural progenitor pools (NPPs), generating primary neurogenesis patterns. In each NPP, several Notch‐independent *Hes/her*‐type genes are expressed in distinct manners. Previous knockdown (KD) experiments induced ectopic neurogenesis in NPPs where only the targeted *her* genes were expressed, with other *her* genes absent, suggesting cooperative functions of Notch‐independent *her* genes. In this study, to overcome the inherent limitations in KD approaches, we knocked out (KO) three *her* genes, *her3*, *her5*, *and her11*, using genome editing techniques. The resulting mutants exhibited ectopic neurogenesis patterns at the end of gastrulation, similar to those observed in KD experiments. KOs of *her5* and *her11* induced ectopic neurogenesis around the midbrain‐hindbrain boundary, whereas *her3* KO led to ectopic neurogenesis in rhombomere 1/2 and r4. In these cases, the expression of other Notch‐independent *her* genes was not affected, except for *her11*, whose expression depended on *her5*. Analyses of compound mutants revealed that their phenotypes were essentially the sum of those of individual *her* mutants, indicating independent suppression of neurogenesis by Notch‐independent *her* genes. In conclusion, different Notch‐independent *her* genes collectively define the characteristic pattern of primary neurogenesis in the neural plate.

## 1 Introduction

In vertebrates, the brain and spinal cord initially develop as the neural plate, which is patterned along the anteroposterior and dorsoventral axes. Subsequently, this neural primordial structure undergoes progressive regionalization and coordinated neuronal differentiation, ultimately giving rise to a highly organized neural network. The neural plate consists of neuroectoderm, which is induced from the dorsal ectoderm through FGF signaling from the posterior and BMP antagonism from the axial mesoderm (Lamb and Harland 1995; Rentzsch et al. 2004; Schier and Talbot 1998). During this process, known as neural induction, neural progenitor cells (NPCs) are specified through the action of neural specifiers, such as *foxd4*, *geminin*, and *soxB1* (Wood and Episkopou 1999; Rex et al. 1997; Rogers et al. 2009; Archer et al. 2011). Neurogenesis is initiated in the neural plate by the activity of bHLH transcription factor (TF) genes, collectively referred to as proneural genes.

Neurogenesis was first elucidated in *Drosophila* and later in vertebrates as being regulated by the interplay among Notch signaling, proneural genes, and *E(Spl)*‐type genes (Beatus and Lendahl 1998; Fehon et al. 1990; Weinmaster 1997; del Diez Corral and Storey 2001). Within proneural clusters, which are defined by proneural gene expression, *delta* genes are activated. The resulting Delta proteins bind to Notch receptors on adjacent cells, leading to the release and nuclear translocation of the Notch intracellular domain (NICD). NICD forms a complex with Su(H)/RBPJ and Mastermind, subsequently activating *Hes/her* genes, which encode E(Spl)‐type bHLH‐O TFs that repress proneural gene expression. This lateral inhibition maintains proneural clusters. Imbalanced lateral inhibition is considered to give rise to neural cells expressing proneural genes and *delta* genes among undifferentiated cells, generating a stable salt‐and‐pepper expression pattern (Kageyama et al. 2008; Matsuda et al. 2015). Recent studies have demonstrated that proneural clusters are maintained as a mixture of neuronally committed and undifferentiated cells through lateral inhibition (Shimojo et al. 2011). The expression of *Hes/her* oscillates due to negative feedback regulation, subsequently leading to the oscillation of proneural genes and *delta* genes. Thus, the salt‐and‐pepper pattern represents a ‘snapshot’ of this dynamic process, with cells in the clusters being maintained in an undifferentiated state (Mumm and Kopan 2000). Hereafter, proneural clusters thus defined will be referred to as proneural cluster domains (PCDs).

The roles of *Hes/her* genes in vertebrate neurogenesis have been intensively evaluated in mice. Major genes include *Hes1, Hes5*, and *Hes7*, whose expression is typically considered Notch‐dependent. However, *Hes1* is also expressed in a Notch‐independent manner in undifferentiated embryonic stem cells (Katoh and Katoh 2007) and in the neural plate. In this context, *Hes1* contributes to the maintenance of the neural progenitor pool (NPP) (Baek et al. 2006). Indeed, the roles of *Hes1* differ depending on its expression pattern. When *Hes1* is stably expressed at high levels, neurogenesis is suppressed, and neural progenitors are maintained. As its expression level decreases, oscillation is initiated due to negative autoregulation. This elicits delayed oscillation of proneural genes and *delta* genes, leading to the maintenance of the progenitor state and proliferation via lateral inhibition. However, once *Hes1* is downregulated, proneural genes are activated, and neural differentiation is promoted, driving the transition from progenitor cells to differentiated neurons (Kageyama et al. 2019).

**FIGURE 1:**
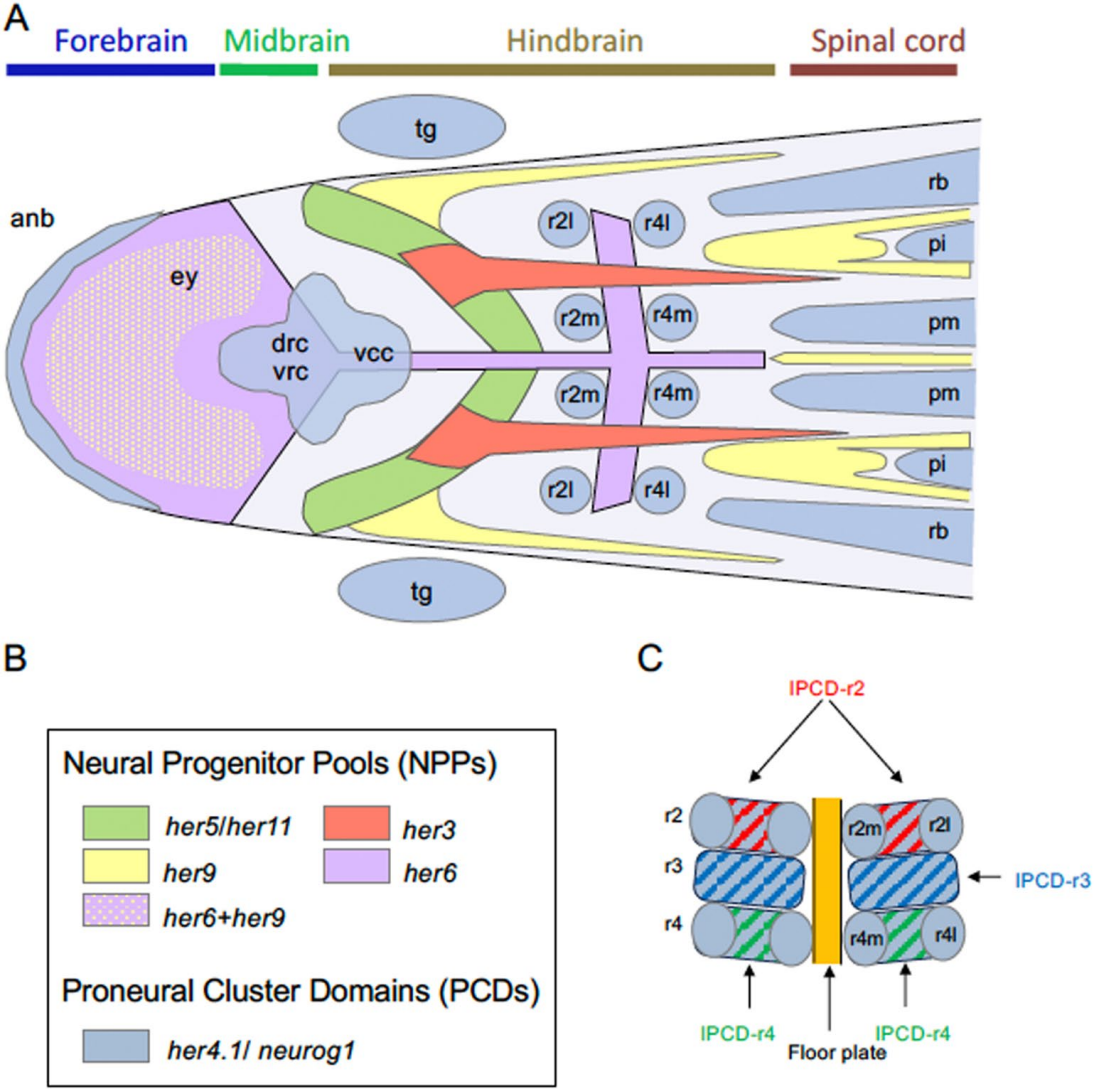
Summary of the neurogenesis pattens in the developing neural plate in zebrafish. (A) Schematic diagram of the anterior neural plate, showing distribution of proneural cluster domains (PCDs) and neural progenitor pools (NPPs) marked by various *her* genes (Stigloher et al. 2008). The expression of *her9* in the floor plate and midbrain‐hindbrain boundary (MHB) region is omitted to avoid confusion (Latimer et al. 2005). Doral views with anterior to the left. anb, anterior neural boundary; drc, dorso‐rostral cluster; ey, eye field; fb, forebrain; mb, midbrain; pi, primary spinal interneurons; pm, primary spinal motor neurons; r2l, lateral neurons in rhombomere 1/2; r2m, motoneurons in rhombomere 1/2; rb, spinal sensory neurons (Rohon‐Beard neurons); tg, trigeminal ganglion; vcc, ventro‐caudal cluster; vrc, ventro‐rostral cluster. (B) Color codes used in panel A. (C) Patterns of PCDs and NPPs in the hindbrain. Distribution of PCDs (r2m, r2l, r4m, r4l) and inter‐PCDs (IPCDs) in the rhombomere 3–5 region (IPCD‐r3–IPCD‐r5) are shown schematically.

In fish such as the zebrafish ( *Danio rerio* ) and amphibians, PCDs are formed in a characteristic pattern in the neural plate, particularly in the hindbrain region, from late gastrulation to early somitogenesis (Chitnis and Dawid 1999; Stigloher et al. 2008) (Figure 1). The earliest neurons arise within these PCDs through primary neurogenesis (Chitnis et al. 1995; Chitnis and Dawid 1999) and enable the escape behaviors necessary for juvenile fish. In the gaps between adjacent PCDs, NPPs are maintained independently of Notch signaling and are preserved for subsequent neurogenesis (Geling et al. 2004), ultimately giving rise to neuronal cells and glial cells in juvenile and adult fish. Importantly, numerous similarities have been observed between NPCs in the developing brain and neural stem cells (NSCs) in the adult brain (Corbin et al. 2008; Imayoshi et al. 2010; Rothenaigner et al. 2011; Alunni and Bally‐Cuif 2016; Caron et al. 2022; Chapouton et al. 2011). Thus, primary neurogenesis in the PCDs and the maintenance of NPPs provide a valuable model system for understanding the regulation of neurogenesis in vertebrates (Stigloher et al. 2008).

Of note, NPPs often function as signaling centers that regulate regionalization of adjacent regions (Geling et al. 2003; Schmidt et al. 2013; Stigloher et al. 2008; Tallafuß and Bally‐Cuif 2003). The anterior neural border (ANB), one of the NPPs, plays a crucial role in controlling the development of the telencephalon and diencephalon (Rubenstein et al. 1998; Houart et al. 2002). The midbrain‐hindbrain boundary (MHB), a well‐characterized NPP, serves as a local organizer that directs the development of the midbrain and cerebellum (Rhinn and Brand 2001; Nakamura 2001; Hidalgo‐Sánchez et al. 2022). Thus, the mechanisms underlying neurogenesis patterns are tightly linked to brain regionalization.

Among zebrafish *Hes/her* genes, *her4.1* depends on Notch signaling and is expressed in a salt‐and‐pepper pattern together with proneural genes and *delta* genes, forming PCDs (Takke et al. 1999; Stigloher et al. 2008; Inomata et al. 2020). By contrast, several *her* genes are expressed independently of Notch at the bud stage, which corresponds to the end of gastrulation (*her3, her5, her9, her11*) (Figure 1A) (Bae et al. 2005; Geling et al. 2003; Hans et al. 2004; Ninkovic et al. 2005; Webb et al. 2011). A series of knockdown (KD) experiments using morpholino oligos (MOs) or *grip*NAs suggested that this group of *her* genes suppresses neurogenesis to maintain NPPs and confine PCD territories (Figure 1A). Indeed, knocking down *her5* and/or *her11*, which are redundantly expressed in the MHB region, led to ectopic neurogenesis in the intervening zone (IZ) between the PCDs in the midbrain and rhombomeres 1/2 (r1/2) (Figure 1A) (Geling et al. 2003, 2004; Ninkovic et al. 2005). Likewise, when mouse *Hes1* was knocked out, the isthmus region was disrupted (Hirata et al. 2001). Knocking down *her3* led to ectopic neurogenesis in the gap regions between lateral (sensory) PCDs and medial (motor) PCDs within r1/2 and r4 (termed r2l/r4l and r2m/r4m, respectively) that express *her3* in wild‐type (WT) embryos (Hans et al. 2004) (referred to as IPCD‐r2 and IPCD‐r4, respectively; IPCD stands for inter‐PCD, Figure 1A,C).

Recently, we assessed the expression and function of *her6*, the zebrafish orthologue of mouse *Hes1* (Davis and Turner 2001), in the neural plate and found that *her6* expression is independent of Notch signaling in the neural plate at the bud stage (Tsuruoka et al. 2025). We used the CRISPR/Cas9 system to disrupt *her6* and examined its roles during neurogenesis. Mutant analyses suggested that *her6* is involved in maintaining several NPPs that were devoid of the expression of other Notch‐independent *her* genes.

However, KD approaches often have inherent limitations, such as off‐target and nonspecific effects, apoptosis induction, uncontrolled or incomplete suppression, and the inability to conduct long‐term phenotypic analysis. These issues may be exacerbated when multiple genes are knocked down in embryos. Thus, these approaches have limitations as tools for precisely understanding gene functions in embryos (Schulte‐Merker and Stainier 2014; Pauli et al. 2015).

In this study, as we did for *her6*, we used genome editing to confirm the roles of other Notch‐independent *her* genes in the patterning of the early neural plate around the end of gastrulation in zebrafish. Furthermore, we examined the interactions among Notch‐independent *her* genes in neural development by analyzing compound mutants. Together, these results enhance our understanding of the patterning functions of Notch‐independent *her* genes in primary neurogenesis.

## 2 Materials and Methods

### 2.1 Animals

Adult zebrafish (*Danio rerio*, RW line) were maintained at 26°C–27°C under a 14‐h light/10‐h dark cycle. Embryos were raised at 28.5°C until appropriate stages. Morphological features as well as hours post‐fertilization (hpf) were used to stage embryos (Kimmel et al. 1995). To suppress pigment formation, 0.2 mM 1‐phenyl‐2‐thiourea (Nacalai Tesque) was used. *her6* mutants were previously generated by the CRISPR/Cas9 method and were employed in this study (Tsuruoka et al. 2025). All experiments using live fish adhered to the protocols approved by the Committee for Animal Care and Use of Saitama University.

### 2.2 Gene Disruption by Genome Editing

**FIGURE 2:**
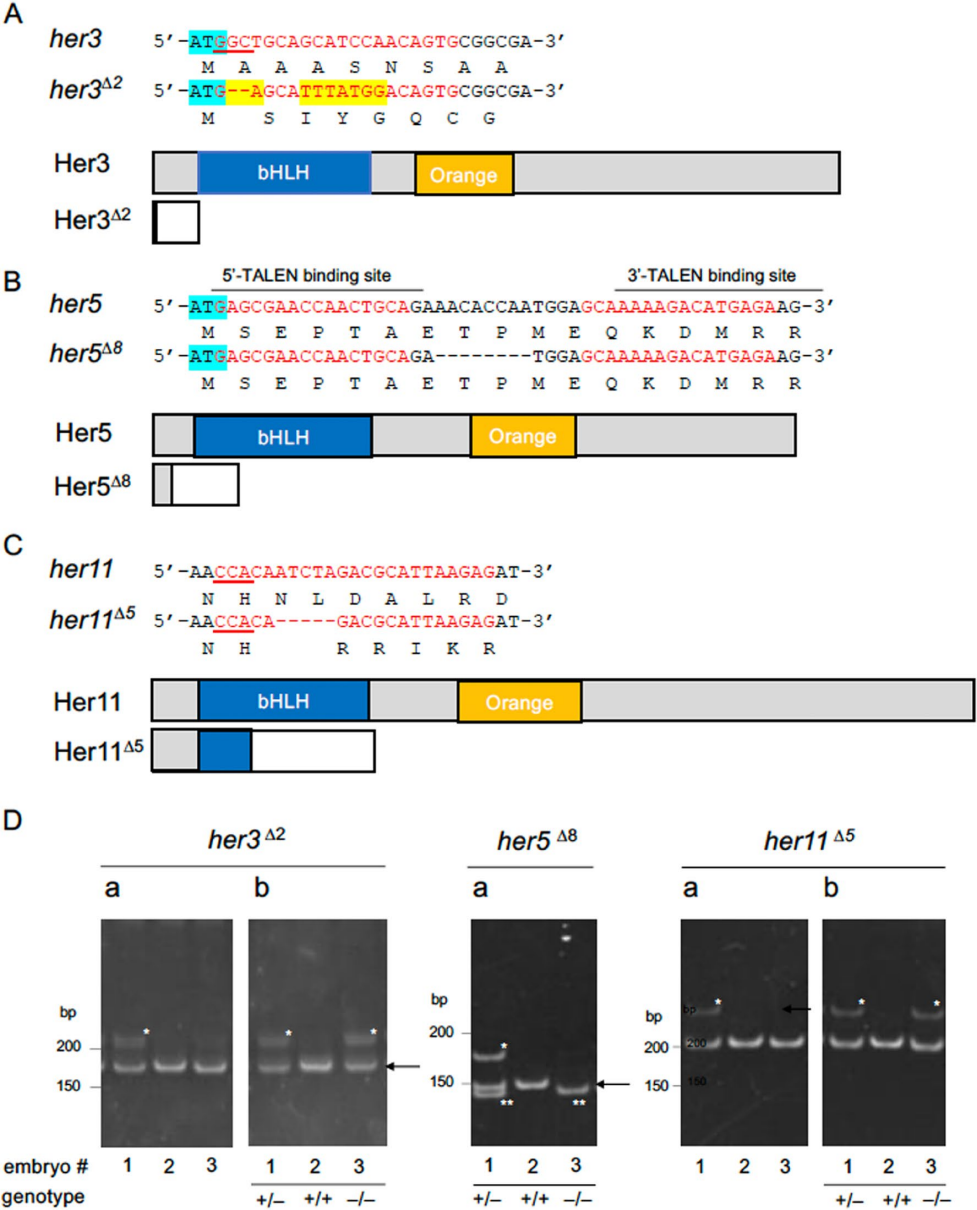
Structures of the mutant *her* genes established in the current study. (A–C) Mutations introduced into Notch‐independent *her* genes by genome editing. Small indels were introduced into the N‐termini (*her3, her5*) or bHLH (*her11*) domains using CRISPR/Cas9 (*her3, her11*) or TALEN (*her5*). The target base sequences (red) and corresponding amino acid sequences are shown above the gene schematics. PAM sequences are underlined, and start codons are marked with light blue. In *her3*, base substitution (yellow) and a small deletion were observed. Wild‐type and disrupted coding sequences due to frameshifts are shown with gray and white boxes, except for bHLH domains and Orange domains that are shown with blue and orange boxes, respectively. In the *her3* mutant line, almost the entire coding region was missing due to a frameshift immediately after the start codon. In *her5* mutants, an 8‐bp deletion resulted in a stop codon at position 29. In the *her11* mutant, a 5‐bp deletion occurred in the N‐terminal portion of the bHLH domain. (D) Genotyping of *her* mutants by the HMA method (examples). Da. Genomic DNA from embryos (#1–#3) was PCR amplified using primer pairs flanking the target sites and analyzed by PAGE. Band shifts (asterisks) indicate heterozygotes, while no shift indicates WT or homozygotes. In *her5* mutants, WT and homozygotes were discriminated by size reduction (double asterisks). For *her3/her11* mutants, PCR products were re‐annealed with WT genomic DNA and analyzed by second PAGE (Db). Band shifts confirm homozygosity.

*her5* was disrupted using the TALEN method. Target sequences for the TALEN pairs were selected using the TAL Effector Nucleotide Targeter 2.0 (https://tale‐nt.cac.cornell.edu/; spacer length, 12 bp–16 bp; repeat array length, 15 bp–20 bp) (cf. Figure 2B). The TALEN constructs were assembled following the two‐step assembly protocol according to the Golden Gate method (Sakuma et al. 2013), using the backbone plasmids (Golden Gate TALEN and TAL Effector Kit 2.0, Addgene) and the Quick Ligation Kit (NEB). mRNA for the TALEN construct pair was synthesized and injected into embryos (200–400 pg/embryo for each arm).

For the N‐terminal or functional domains of other *her* genes (*her3* and *her11*), gRNAs were designed using CRISPRdirect (https://crispr.dbcls.jp/) (cf. Figure 2A,C) (Yuikawa et al. 2024) and synthesized using the CUGA7 gRNA Synthesis Kit (Nippon Gene). The gRNA was co‐injected with Cas9 protein (Nippon Gene) into fertilized eggs (20 pg/embryo and 800 pg/embryo, respectively).

Disruption of the target sequences was confirmed by the heteroduplex mobility assay (HMA) (Ota et al. 2013) using primer pairs flanking the target sites (Table S1), followed by direct sequencing of the target sites amplified by polymerase chain reaction (PCR) using extracted genomic DNA as templates.

### 2.3 Genotyping of Mutants by the HMA Method

For genotyping, genomic DNA was extracted individually from live or stained embryos, or from adult fish, and subjected to sequential HMA using the same primer pairs mentioned above to discriminate WT, heterozygotes, and homozygotes (Umeda et al. 2024). Alternatively, when the sizes of indels were sufficiently large, WT‐homozygote discrimination was possible simply by the size difference of the PCR products.

### 2.4 Staining of Embryos

Riboprobes labeled with digoxigenin (DIG) were synthesized with the DIG RNA Labeling Mix (Roche Diagnostics), using T3 or T7 RNA polymerase (Stratagene), according to the manufacturers' protocols. Whole mount in situ hybridization (WISH) was conducted as described previously (Kikuta et al. 2003).

### 2.5 Microscopic Observation

Live and stained embryos were observed under a fluorescence stereomicroscope (MZFLIII, Leica), with images captured using a cooled CCD camera (DFC 300 FX, Leica) and Leica Application Suite Version 3.3.1 (Leica).

## 3 Results

### 3.1 Disruption of Notch‐Independent *her* Genes for Reevaluation of the Previous KD Experiments

The roles of Notch‐independent *her* genes in the developing neural plate were previously addressed using KD approaches, but these approaches often produce artifacts and have limitations in their application. In the current study, to confirm and extend the findings of the KD‐based experiments, three *her* genes—*her5*, *her3*, and *her11*—were disrupted by genome editing techniques. Frameshifts were introduced at the N‐terminal ends (*her3, her5*) or in the bHLH domain (*her11*) using the TALEN method (*her5*) or CRISPR/Cas9 method (*her3, her11*), resulting in small base deletions (*her5, her11*) or both deletion and substitution (*her3*). In the *her3* mutant, almost the entire coding region was disrupted. In *her5* mutants, the gene product also lacked most of the sequence, including the entire bHLH domain. The *her11* mutant was predicted to lack the C‐terminal two thirds of the bHLH domain and the following sequence. Thus, these mutants were expected to be functionally null (*her3* ^*∆2*^, *her5* ^*∆8*^, *her11* ^*∆5*^; Figure 2).

### 3.2 Phenotype Analyses of *her5* Mutants

**FIGURE 3:**
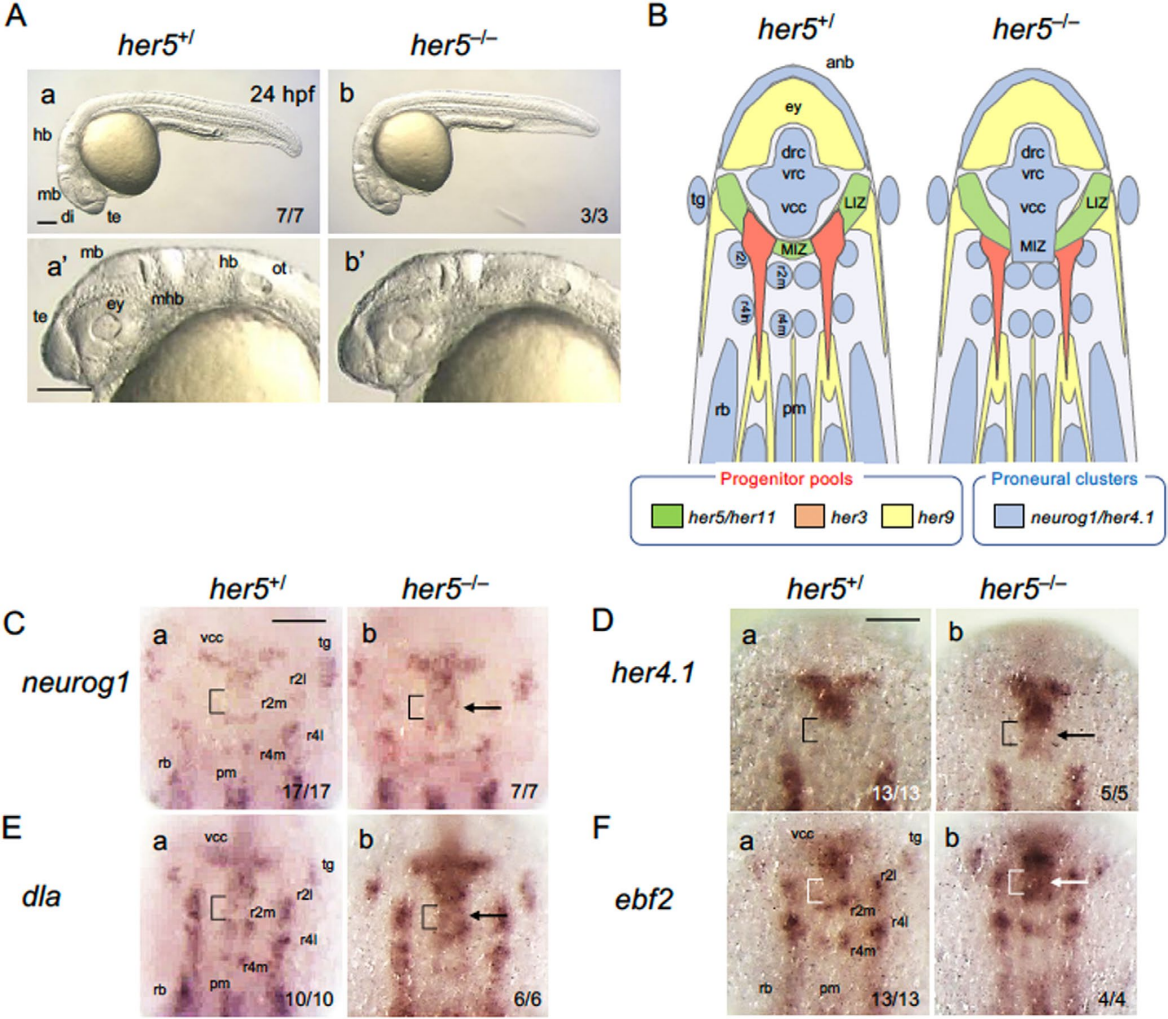
Phenotypes of *her5* mutants in the developing brain. (A) Morphology of *her5* mutants at 24 hpf. Live embryos from heterozygous mating (*her5* ^*+/Δ8*^) were photographed and genotyped. Lateral views of whole embryos (a, b) and heads (a′, b′) are shown with anterior to the left and dorsal to the top. Numbers of embryos with each phenotype and total scored embryos are indicated. Scale bars, 200 μm. (B) Schematic comparison of PCD gene expression in wild‐type (WT, left) and *her6* homozygotes (right). Dorsal views (anterior top). (C–F) Expression of proneural cluster domain (PCD)‐related genes in *her5* mutants. WISH staining was performed at the bud stage, followed by imaging and genotyping. Dorsal views of the midbrain‐hindbrain region are shown with anterior to the top; whole‐embryo views are shown in Figure S2A. Arrows mark ectopic expression in MIZ (brackets). Numbers of the embryos with the indicated expression patterns and total scored embryos are shown in the bottom‐right. *her5* ^*+/+*^ and *her5* ^*+/−*^ embryos showed identical phenotypes and were grouped as *her5* ^*+/*^, and scored accordingly; images show WT (*her5* ^*+/+*^) embryos as representatives. Scale bars, 100 μm. di, diencephalon; hb, hindbrain; LIZ, lateral intervening zone; mhb, midbrain‐hindbrain boundary; mb, midbrain; MIZ, medial intervening zone; ot, otocyst; ov, optic vesicle; te, telencephalon. For the remaining abbreviations, see the legends to Figures 1 and 3.

**FIGURE 4:**
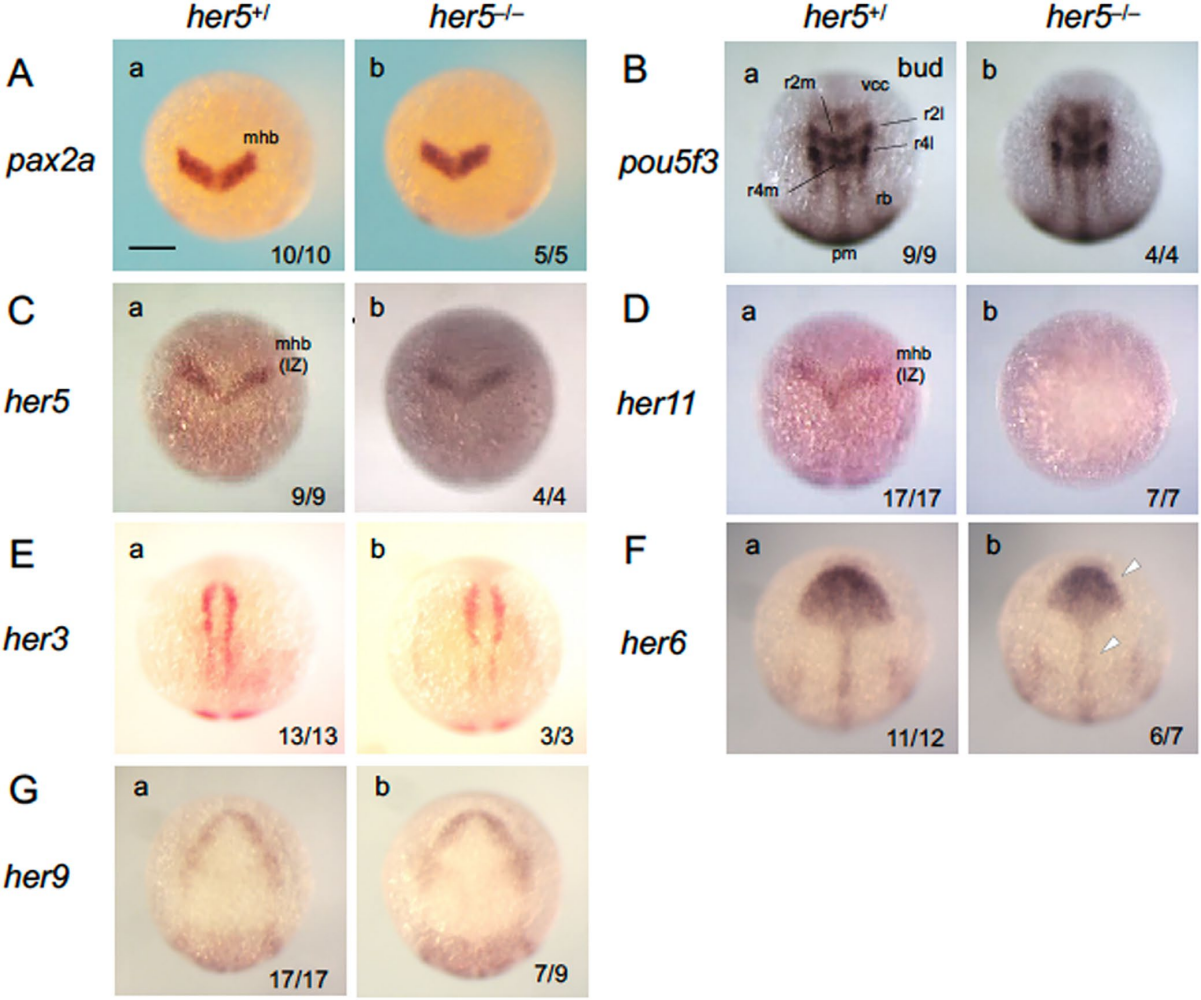
Expression of brain regionalization genes and *her* genes in *her5* mutant embryos. Embryos from heterozygous mating (*her5* ^*+/Δ8*^) were examined for the expression of MHB‐forming genes and *her* genes at the bud stage by WISH, followed by genotyping. Open arrowheads show weak downregulation. See the legends to Figures 1 and 3 for abbreviations. Numbers of the embryos with the indicated expression patterns and total scored embryos are shown in the bottom‐right. *her5* ^*+/−*^ embryos were indistinguishable from *her5* ^*+/+*^ embryos. Thus, both were scored together and referred to as *her5* ^*+/*^. Images for *her5* ^*+/+*^ embryos are shown as representatives. Scale bar, 200 μm.

We first assessed the phenotypes of *her5* mutants, as KD experiments have been extensively conducted for this *her* gene (Geling et al. 2003, 2004; Ninkovic et al. 2005). Offspring from heterozygous mating (*her5* ^*+/Δ8*^) were subjected to phenotype analyses. Morphologically, both homozygotes and heterozygotes showed no anomalies at 24 hpf, even around the isthmus (Figure 3A; “+/” represents a combined genotype, including “+/+” and “+/−”, and is used when heterozygotes show no anomalies.). The isthmus, formed at the MHB where *her5* is expressed at earlier stages, developed normally, and the fish grew into fertile adults. At the bud stage, *her5* expression (Müller et al. 1996) separates the vcc and r1/2 PCDs (Figure 1A), and KD experiments suggested that *her5* is essential for establishing the medial portion of the IZ (MIZ) through suppression of neurogenesis (Geling et al. 2003, 2004; Ninkovic et al. 2005). We assessed the neurogenesis pattern in the neural plate at the bud stage by WISH in embryos from heterozygous mating. The proneural gene *neurog1* was ectopically activated only in homozygotes in the medial MHB region, which lacked *neurog1* expression in WT and heterozygotes (*her5* ^*+/*^), corresponding to the MIZ defined by KD experiments (Figure 3B,C, Figure S1A). Abnormal *neurog1* expression was never observed in heterozygotes. *neurog1* expression was normal in other regions of homozygotes (Figure S1A). In contrast, *neurog1* expression was nearly normal at 24 hpf in the entire brain of homozygotes, indicating recovery of neurogenesis(Figure S2A–D). These observations are essentially consistent with the findings obtained in the KD studies. We also observed ectopic expression of *her4.1*, *deltaA* (*dla*), and *ebf2* in the MIZ in homozygotes alone (Figure 3B,D–F; Figure S1B–D). These observations indicated that the *her5* ^*∆8*^ mutation is recessive in neurogenesis patterning in the neural plate. Notably, in *her5* mutants, the expression of the MHB marker *pax2a* was normal at both the bud stage and 24 hpf, indicating correct MHB establishment (Figure 4A, Figure S2E,F). The expression of *pou5f3*, which is required for MHB establishment (Belting et al. 2001; Burgess et al. 2002) and neurogenesis in the hindbrain (Hauptmann et al. 2002; Inomata et al. 2020), was normal (Figure 4B), supporting proper MHB establishment even in *her5* homozygotes.

We also examined the expression of *her11*, a paralogue of *her5*, which is located 3.3 kb upstream of *her5* (Ninkovic et al. 2005). Like *her5*, *her11* is expressed in the MHB and paraxial mesoderm (Ninkovic et al. 2005). In a previous study, *her5* KD did not affect *her11* expression, and both genes were suggested to suppress neurogenesis in parallel in a dose‐dependent manner (Ninkovic et al. 2005). However, in *her5* homozygotes, but not in *her5* heterozygotes, *her11* expression was disrupted in the MHB region and presomitic mesoderm (PSM) from 80% to 24 hpf (Figure 4D, Figure S2G–N). This excludes the possibility that the discrepancy is due to differences in developmental stages, suggesting that *her11* continuously requires *her5* for its expression. In contrast, *her5* expression remained normal in *her5* mutant embryos, indicating the absence of autoregulation (Figure 4C).

The expression of other Notch‐independent *her* genes–*her3*, *her6*, and *her9*–was also assessed in *her5* mutants (Figure 4E–G), with no evident anomalies, although *her6* showed slight downregulation in homozygotes alone. Thus, the expression of these genes appears to be essentially independent of *her5*.

### 3.3 Phenotype Analyses of *her11* Mutants

**FIGURE 5:**
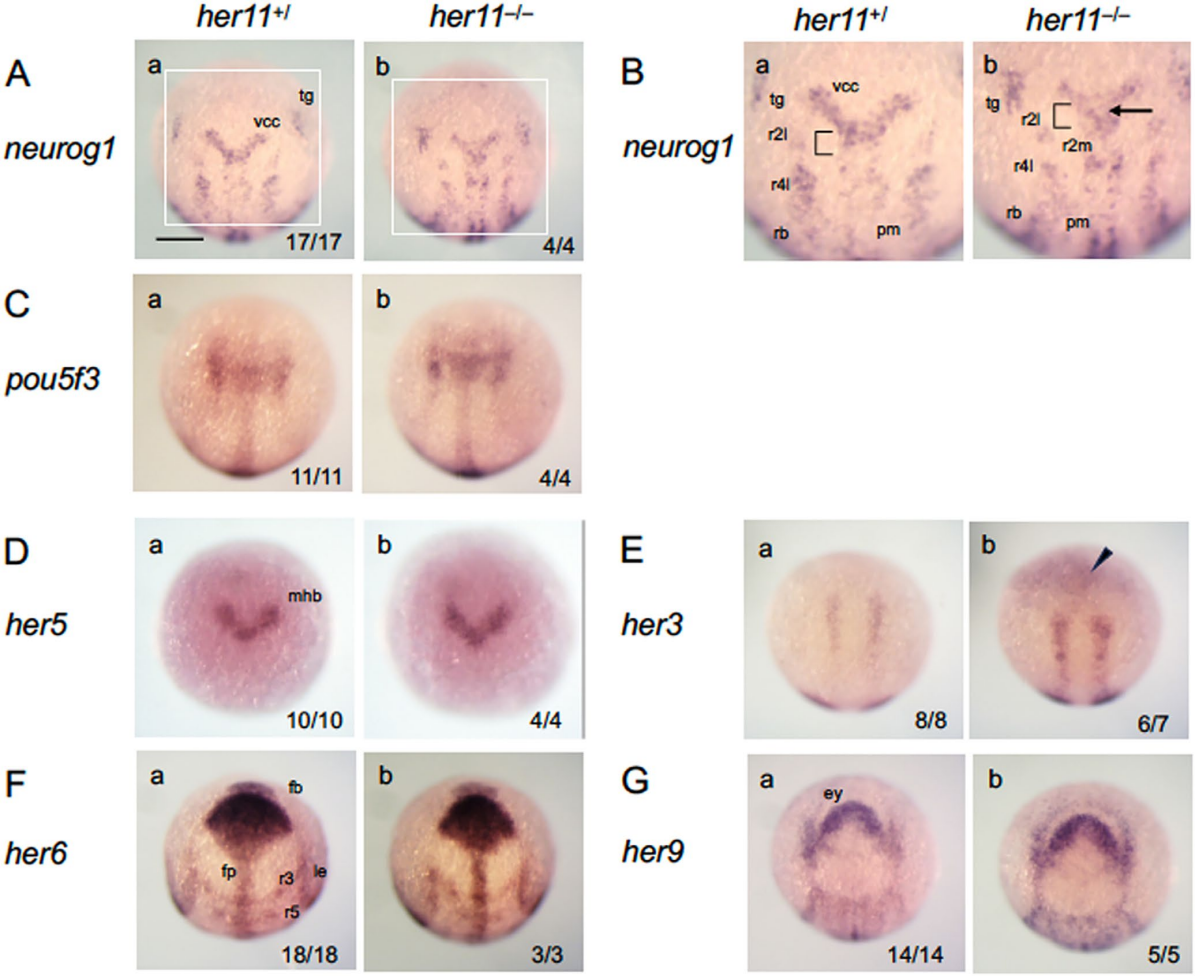
Phenotypic analysis of *her11* mutant embryos. Embryos from heterozygous mating (*her11* ^*+/Δ5*^) were examined for the expression of *neurog1* (A, B), *pou5f3* (C), and *her* genes (D–H) by WISH and photographed, followed by genotyping. A, B. For *neurog1* expression, boxed areas in A are enlarged in B. Arrows mark ectopic expression in MIZ (brackets). Solid arrowheads in B show weak upregulation. See the legends to Figures 1 and 3 for abbreviations. Numbers of the embryos with the indicated expression patterns and total scored embryos are shown in the bottom‐right. *her11* ^*+/+*^ and *her11* ^*+/−*^ embryos showed identical phenotypes and were grouped as *her11* ^*+/*^, and scored accordingly; images show wild‐type (*her11* ^*+/+*^) embryos as representatives. Scale bars, 200 μm.

We further evaluated the phenotypes of the *her11* mutants established here ( *her11* ^*Δ5*^ ) (Figure 5). Both *her11* heterozygotes and homozygotes developed normally and grew to adulthood, similar to *her5* mutants (data not shown). Previous studies showed that neurogenesis genes were ectopically activated by *her11* KD in the MIZ (Ninkovic et al. 2005), and this was recapitulated in *her11* homozygotes alone (Figure 5A,B). As with *her5* mutants, we observed that *pou5f3* expression was unaffected, (Figure 5C). Previous *her11* KD did not affect *her5* expression. Consistent with this, *her5* expression was normal even in *her11* homozygotes (Figure 5D), confirming that *her5* expression is independent of *her11*. Interestingly, *her3* expression was upregulated in its normal domain, and weak ectopic expression was observed broadly in the anterior domain of most homozygotes, but not in heterozygotes. This suggests global repression of *her3* by *her11* (Figure 5E). In contrast, the expression of other Notch‐independent *her* genes, *her5*, *her6*, and *her9*, remained unaffected (Figure 5F,G).

### 3.4 Assessment of Neurogenesis in *her3* Mutants

*her3*, the orthologue of mouse *Hes3*, is expressed during early somitogenesis in the neural plate as two longitudinal stripes that extend posteriorly from the MHB, located between the lateral (Sensory) and medial (Motor) PCDs (Figure 1A). KD of *her3* caused ectopic expression of *neurog1* in the IPCD‐r2 and IPCD‐r4 (Figure 1C). Furthermore, Her3 is considered to repress its own transcription (Hans et al. 2004). In the current study, we attempted to re‐assess the reproducibility of the KD experiment using newly established *her3* mutants (*her3* ^*+/*^ ^*Δ2*^ ). Morphologically, no noticeable abnormalities were observed even in *her3* homozygotes at 24 hpf (data not shown), which later grew to fertile adult fish.

**FIGURE 6:**
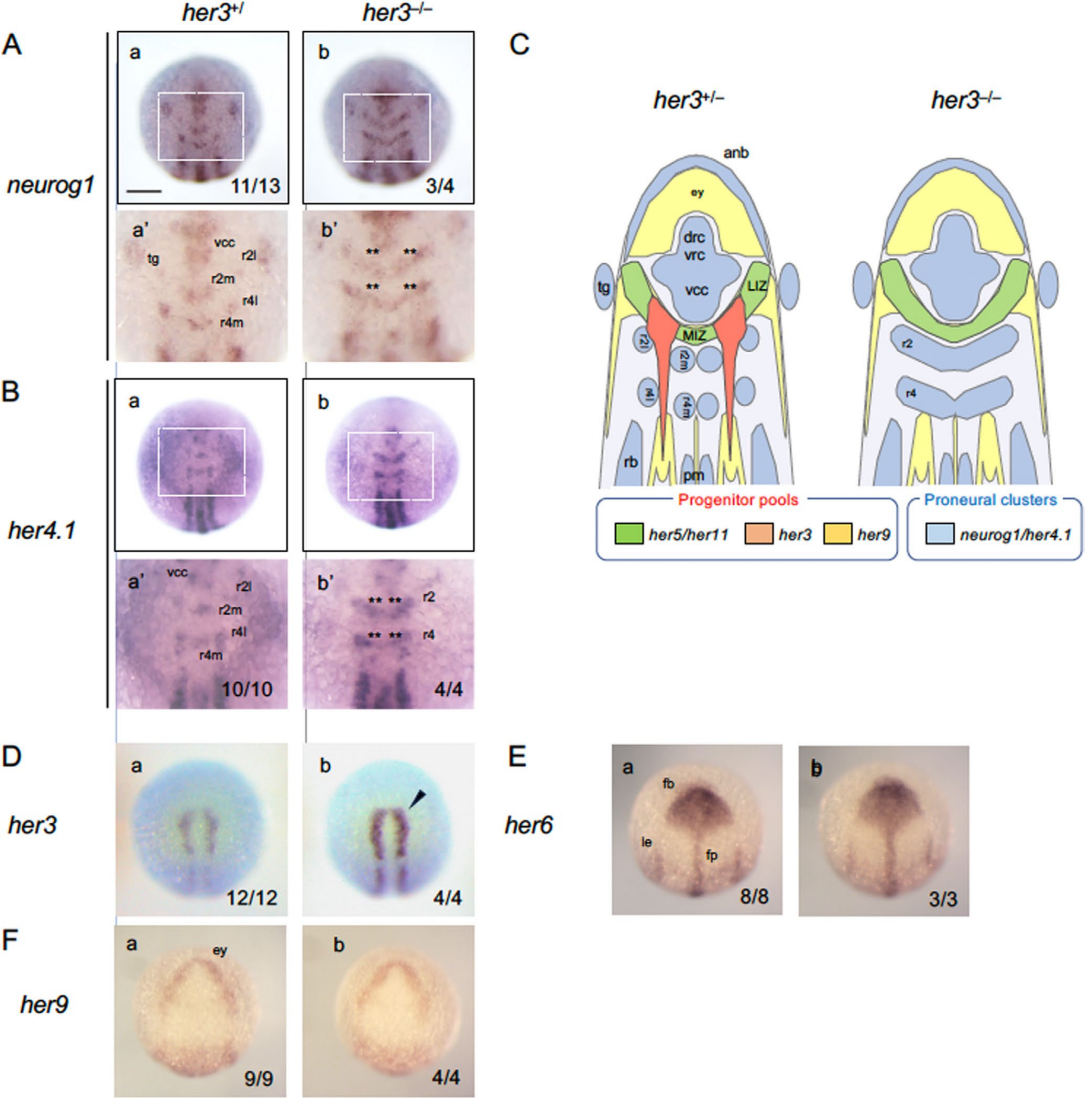
Phenotypic analysis of *her3* mutant embryos. (A) Embryos from heterozygous mating (*her3* ^*+/Δ2*^) were examined for the expression of neurogenesis‐related (A, B) and Notch‐independent *her* (D–G) genes by WISH, followed by genotyping. For each gene, boxed areas are enlarged below the whole views. Double asterisks (**) indicate ectopic expression in IPCD‐r2/IPCD‐4. Solid arrowheads show weak upregulation. Numbers of the embryos with the indicated expression patterns and total scored embryos are shown in the bottom‐right. *her3* ^*+/+*^ and *her3* ^*+/−*^ embryos showed identical phenotypes and were grouped as *her3* ^*+/*^, and scored accordingly; images show wild‐type (WT, *her3* ^*+/+*^) embryos as representatives. Scale bars, 200 μm. (C) Schematic diagram, showing normal and ectopic neurogenesis observed in the IPCD in r1/2 (IPCD‐r2) and in r4 (IPCD‐r4) in WT embryos and *her3* homozygotes, respectively. See the legends to Figures 1 and 3 for abbreviations.

**FIGURE 7:**
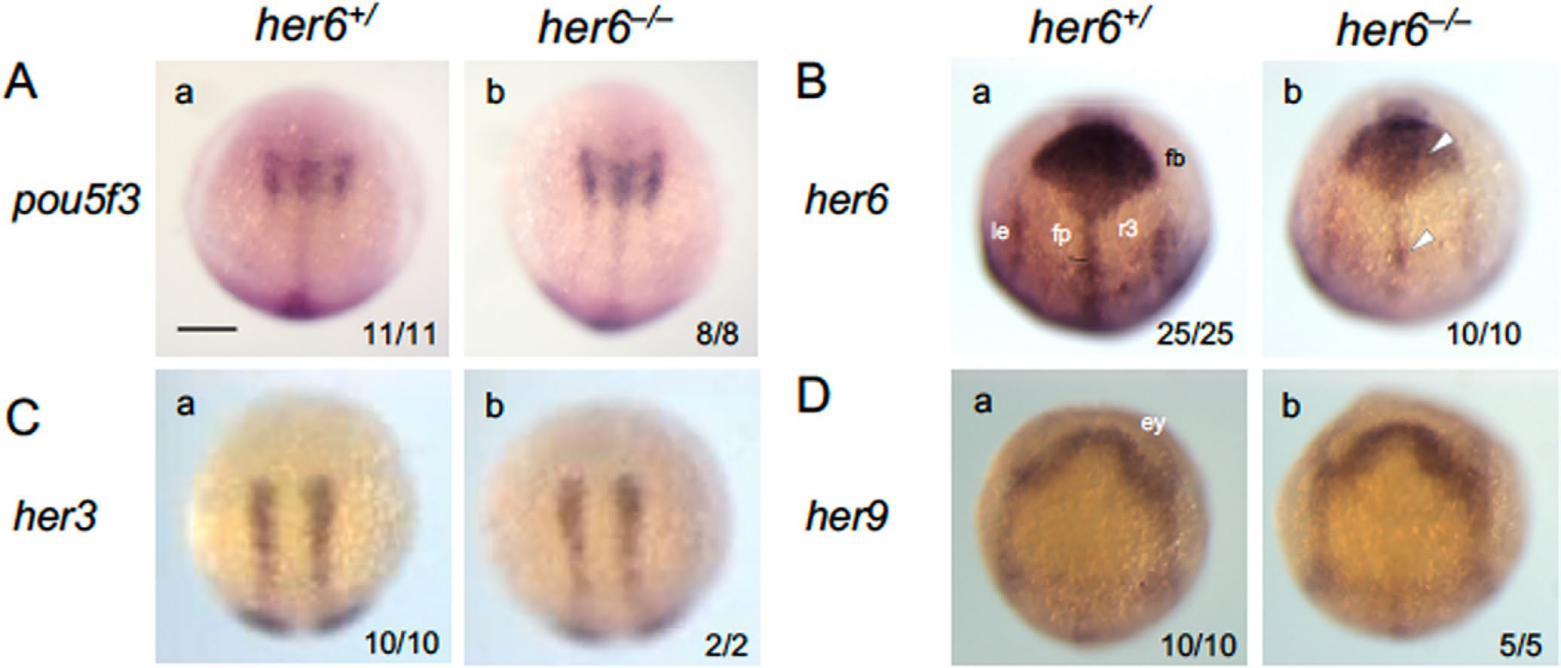
Expression of Notch‐independent *her* genes in *her6* mutants. Embryos obtained by heterozygous mating of *her6* mutants (*her6* ^*+/Δ5*^) were examined for the expression of Notch‐independent *her* genes by WISH, followed by genotyping. Open arrowheads show weak downregulation. Numbers of the embryos with the indicated expression patterns and total scored embryos are shown in the bottom‐right. *her6* ^*+/−*^ embryos were indistinguishable from *her6* ^*+/+*^ embryos, and both were scored together and referred to as *her6* ^*+/*^. Images for wild‐type (*her6* ^*+/+*^) embryos are shown as representatives. fp, floor plate; le, lateral ectoderm. See the legends to Figures 1 and 3 for other abbreviations. Scale bars, 200 μm.

The expression of *neurog1* and *her4.1* was examined in mutants at the bud stage. Importantly, *neurog1* was ectopically expressed in homozygotes, but not in heterozygotes, between motor neuron and sensory neuron progenitors in both r1/2 and r4 (IPCDs‐r2, ‒r4), where NPPs are present in WT embryos (Figure 6A,C). This ectopic expression is consistent with the defects previously observed in *her3* KD experiments (Hans et al. 2004). Ectopic expression was not observed in the posterior neural plate (Presumptive Spinal Region) in *her3* homozygotes, also reproducing the KD results. Essentially the same anomaly was obtained for *her4.1* expression in homozygotes alone (Figure 6B). Compared to WT and heterozygous embryos, the *her3* expression pattern was normal but upregulated in homozygotes, suggesting that *her3* is regulated by a negative autoregulatory loop (Figure 6D). The expression of other Notch‐independent *her* genes, *her6* and *her9*, was not evidently affected (Figures 6F,G and 7).

### 3.5 The Absence of *her6* Did Not Affect the Expression of Other Notch‐Independent *her* Genes

**FIGURE 8:**
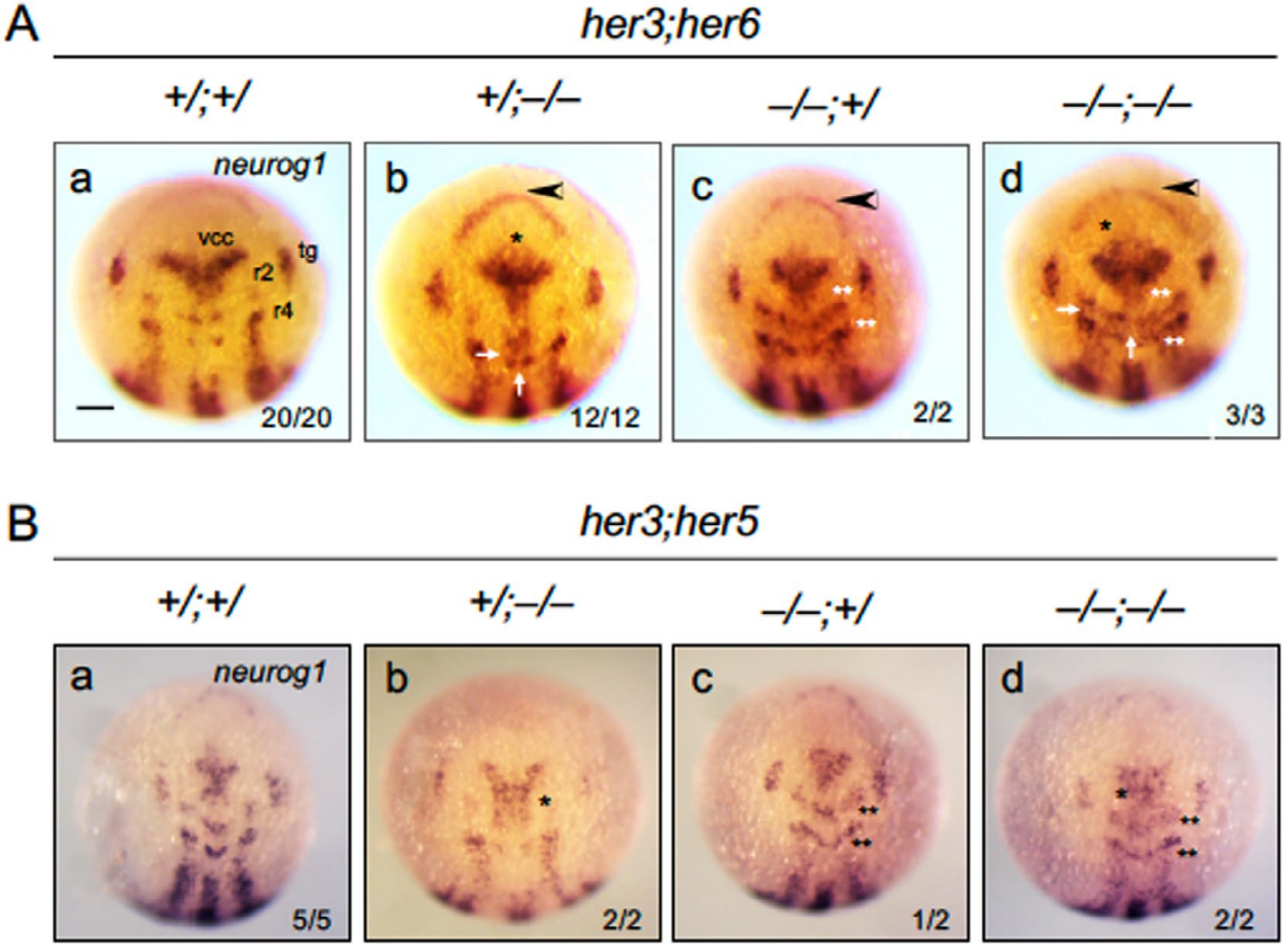
Abnormal neurogenesis in *her* compound mutants revealed by the expression of *neurog1*. The expression of *neurog1* was examined by WISH in embryos from mating between *her3/her6* double heterozygote fish (A, *her3* ^*+/Δ2*^; *her6* ^*+/Δ5*^) or *her3/her5* double heterozygote fish (B, *her3* ^*+/Δ2*^; *her5* ^*+/Δ8*^) at the bud stage, photographed, and then genotyped. Dorsal views with anterior to the top. Numbers of embryos with the indicated expression patterns and the numbers of total scored embryos are shown in the bottom right. See the text for the symbols, ‘+/;+/’, ‘+/;−/−’, ‘−/−;+/’, and ‘−/−;−/−’). (A) Arrowheads indicate increased expression in the anterior‐most portion of the neural plate, thin horizontal arrows indicate ectopic expression in the r3 region (m‐IPCD‐r3), thick vertical arrows mark md‐IPCD‐r3/5, and single asterisks (*) indicate ectopic expression around drc and vrc. Ectopic expression in IPCD‐r2/IPCD‐r4 is indicated by double asterisks (**). (B) Ectopic expression in MIZ and in IPCD‐r2/IPCD‐4 is marked with single and double asterisks, respectively. For abbreviations, see the legends to Figures 1 and 3. Scale bar, 200 μm.

We previously showed, using *her6* mutants, that this *her* gene works to maintain NPPs in the forebrain, midbrain, and hindbrain (Tsuruoka et al. 2025) (cf. Figure 8). To clarify whether *her6* regulates the expression of other Notch‐independent *her* genes, we assessed the expression of these genes in *her6* mutants. First, the expression of *her6* itself was normal regarding the pattern, but the intensity was decreased in all regions in homozygotes compared to WT and heterozygous embryos (*her6* ^*+/*^, Figure 7B). This suggests the presence of a negative autoregulatory loop for *her6*, although the involvement of the quality control mechanism for mRNA, such as nonsense‐mediated mRNA decay (NMD), is possible. Meanwhile, the expression of *her9*, which overlaps with that of *her6* in the forebrain, and the expression of *her3*, which partially overlaps with *her6* expression in r3, were normal in *her6* mutants (Figure 7C,D), suggesting that *her3* and *her9* are expressed independently of *her6*. The expression of *pou5f3* was normally observed in *her6* mutants as in the mutants for other *her* genes (Figure 7A).

### 3.6 Interactions Among Notch‐Independent *her* Genes During Neurogenesis in the Neural Plate

We further examined genetic interactions among Notch‐independent *her* genes in neurogenesis by analyzing *neurog1* expression in compound mutants. First, we crossed *her3/her6* double heterozygote fish (*her3* ^*+/−*^ *; her6* ^*+/−*^) and examined *neurog1* expression in their offspring (Figure 8A). In mutants where either or both of the *her* mutations were heterozygous (*her3* ^*+/+*^; *her6* ^*+/−*^, *her3* ^*+/−*^; *her6* ^*+/+*^, *her3* ^*+/−*^; *her6* ^*+/−*^), *neurog1* expression was essentially normal compared to WT (*her3* ^*+/+*^; *her6* ^*+/+*^) embryos (Figure 8Aa). Thus, these embryos were treated as a single genotype (+/;+/) and scored accordingly. In *her6* homozygotes (*her3* ^*+/*^; *her6* ^*−/−*^), which included both *her3* ^*+/+*^; *her6* ^*−/−*^ and *her3* ^*+/−*^; *her6* ^*−/−*^ *embryos* (+/;−/−, Figure 8Ab), ectopic *neurog1*expression was detected in the anterior neural boundary (anb) (Houart et al. 2002), dorso‐rostral cluster (drc) and ventro‐rostral cluster (vrc) in the forebrain, ventro‐caudal cluster (vcc) in the midbrain (Ross et al. 1992), the medial portion of the IPCD region in r3 (IPCD‐r3) between r2m and r4m (m‐IPCD‐r3), and the midline of the r3‐r5 region (md‐IPCD‐r3/5), all of which corresponded to IPCDs present in the neural plate at this stage (Figure 1A). These ectopic expression patterns closely matched those previously observed for *her6* homozygotes (Tsuruoka et al. 2025), regardless of the *her3* genotype. In *her3* homozygotes (*her3* ^*−/−*^ *; her6* ^*+/*^), including *her3* ^*−/−*^; *her6* ^*+/+*^ and *her3* ^*−/−*^; *her6* ^*+/−*^, ectopic expression was again observed in IPCD‐r2 and IPCD‐4, irrespective of the *her6* genotype (−/−;+/, Figure 8Ac). Importantly, in double homozygotes (*her3* ^*−/−*^; *her6* ^*−/−*^), all these ectopic/enhanced expression patterns were observed with no additional anomalies (−/−;−/−, Figure 8Ad). Thus, the phenotypes of double homozygotes appeared to be a simple sum of those of single homozygotes, suggesting that *her3* and *her6* independently contribute to neurogenesis patterning.

We further examined offspring embryos from crosses of *her3* ^*+/−*^; *her5* ^*+/−*^ fish, revealing a similar summation of the two phenotypes (Figure 8B). In “*her3* ^*+/*^; *her5* ^*+/*^” embryos, where either or both of the *her* mutations were heterozygous (*her3* ^*+/+*^; *her5* ^*+/−*^, *her3* ^*+/−*^; *her5* ^*+/+*^, *her3* ^*+/−*^; *her5* ^*+/−*^), the *neurog1* expression patterns were indistinguishable from those in WT embryos (*her3* ^*+/+*^; *her5* ^*+/+*^), and thus, these were regarded as a single genotype (+/;+/, Figure 8Ba). Ectopic expression of *neurog1* was detected in the MIZ of *her3* ^*+/+*^; *her5* ^*−/−*^ and *her3* ^*+/−*^; *her5* ^*−/−*^ embryos, as observed in *her5* ^*−/−*^ embryos, regardless of the *her3* genotype (+/;−/−, Figure 8Bb). The *her3* ^*−/−*^; *her5* ^*+/*^ mutation, including both *her3* ^*−/−*^; *her5* ^*+/+*^ and *her3* ^*−/−*^; *her5* ^*+/−*^, resulted in ectopic expression in IPCDs‐r2 and ‐r4, similar to that in *her3* homozygotes, regardless of the presence of the *her5* mutation (−/−;+/, Figure 8Bc). In double homozygotes, all these ectopic expression patterns were observed again without any additional abnormal expression (−/−;−/−, Figure 8Bd).

## 4 Discussion

The roles of Notch signaling and *her* family genes in the patterning of the neural plate were examined in early zebrafish embryos, in which primary neurogenesis takes place to generate characteristic patterns of neural development. In this first phase of neurogenesis, PCDs are maintained by a salt‐and‐pepper mode of neurogenesis gene expression. Importantly, PCDs exhibit a characteristic spotted pattern in the early neural plate (Figure 1A), where respective PCDs are surrounded by NPPs. Several studies employing KD approaches have suggested that Notch‐independent *her* genes maintain NPPs that are set aside for later neurogenesis (Stigloher et al. 2008; Schmidt et al. 2013). Thus, primary neurogenesis in early zebrafish embryos provides an excellent yet simple model system for understanding the emergence and maintenance of PCDs and NPPs, subsequent regulation of neurogenesis in later development, and NSC maintenance and neuroregeneration in the adult brain. However, KD experiments suffer from numerous limitations, and caution should be taken when interpreting the results, as discussed in the previous sections.

### 4.1 Validation of Previous KD Experimental Findings Through Gene Disruption

To overcome the limitations of KD experiments and obtain more reliable insights into gene functions, we employed genome editing to generate mutants. Indeed, we recently established *her6* mutants, which support the idea that Notch‐independent *her* genes pattern primary neurogenesis in the neural plate (Tsuruoka et al. 2025). In the current study, we generated mutants for other representative Notch‐independent *her* genes and reassessed their phenotypes at the bud stage, when characteristic primary neurogenesis patterns emerge.

Upon KD of *her5* and/or *her11*, ectopic neurogenesis was observed in the MIZ between the PCDs in the midbrain and r1/2 (Geling et al. 2004; Ninkovic et al. 2005). In *her5* ^*Δ8*^ homozygotes, we observed that two proneural genes, *neurog1* and *ebf2*, along with *her4.1* and *dla*, were ectopically activated specifically in the MIZ, whereas their expression in different PCDs, such as vcc, r2m/r2l, and r4m/r4l, remained normal. These findings verify that *her5* suppresses neurogenesis in the MIZ (Figure 3B), consistent with previous KD experiments. The expression of *pax2a* and *pou5f3* remained unchanged, indicating that ectopic neurogenesis in the MIZ was not caused by misspecification of the MHB region.

*her11* and *her5* are co‐orthologues of mouse *Hes7* (Ninkovic et al. 2005). *her11* is co‐expressed with *her5* in the IZ, and both have been suggested to play equivalent roles in IZ formation. Indeed, previous KD of *her11* induced ectopic expression of *neurog1* in the MIZ, similar to the effect of *her5* KD. In the current study, similar ectopic expression was likewise observed in *her11* mutants, further supporting the conclusions drawn from KD experiments. Notably, double KD led to a lateral expansion of ectopic *neurog1* expression, identifying the lateral portion of the IZ (LIZ) as NPPs. Further analyses using *her5/her11* double homozygotes are required.

Upon *her3* KD, ectopic neurogenesis occurred in the NPPs of r1/r2 and r4, between the lateral and medial PCDs (IPCD‐r2 and IPCD‐r4, respectively) (Hans et al. 2004) (Figure 1C). These observations were further confirmed in this study using *her3* mutants (Figure 6C). In homozygous embryos, ectopic expression of *neurog1* and *her4.1* was restricted to IPCD‐r2/r4, where *her3* is the only Notch‐independent *her* gene expressed. This supports the idea that *her3* consistently suppresses neurogenesis, thereby forming bilateral longitudinal NPP stripes.

### 4.2 Regulatory Interaction Among Notch‐Independent *her* Genes

We further assessed the expression dependency among Notch‐independent *her* genes to determine whether mutual regulatory interactions exist. In *her5* and *her11* homozygotes, the expression of *her6*, *her3*, and *her9* was largely unaffected, with only marginal weakening of *her6* in *her5* KO embryos. The expression patterns of these *her* genes (Pasini et al. 2001; Tsuruoka et al. 2025; Bae et al. 2005; Hans et al. 2004) partially overlap with those of *her5* and *her11* (Ninkovic et al. 2005), particularly in the MHB and, in the case of *her6*, in the floor plate (Figure 1A). These observations suggest that these Notch‐independent *her* genes function independently of *her5* and *her11*. Upregulation of *her3* in the original expression domains and anterior neural plate in *her11* mutants was notable, which may result from global negative regulation of *her3* by *her11*, although whether this regulation is direct or mediated by other regulatory factors remains unclear. The expression of *her5* was normal in *her5* homozygotes, suggesting the absence of an autoregulatory loop for this gene, as previously indicated by KD experiments. *her5* expression was also unaffected in *her11* mutants (Ninkovic et al. 2005). Unexpectedly, *her11* expression was completely lost in *her5* mutants in the MHB and PSM from 80% epiboly to 24 hpf, contradicting the findings of the same KD study.

There are two possible hypotheses regarding this contradiction. The first is straightforward; *her11* is positively regulated downstream. In this case, it is possible that KD did not fully suppress *her5* expression. As Her proteins are generally transcriptional repressors, Her5 may indirectly upregulate *her11* by repressing genes that act to suppress its expression. The second hypothesis proposes that the deleted site in *her5* ^*Δ8*^ mutants is a cis‐regulatory element (CRE) required for *her11* expression during neurogenesis. *her11* and *her5* are closely positioned on the same chromosome (Chr 14), approximately 2 kb apart in opposite directions. Additionally, the intergenic 3.3‐kb region that recapitulates the *her5* expression pattern in the MHB region includes exon 1 of *her11* (Ninkovic et al. 2005; Tallafuß and Bally‐Cuif 2003). Therefore, it is possible that the *her11* CRE extends into the *her5* gene, and the deletion in *her5* ^*Δ8*^ mutants could disrupt the CRE required for *her11* expression.

However, given that the disrupted site is within the coding sequence, we consider the first hypothesis more likely. To elucidate how Her5 regulates *her11*, it will be necessary to examine the effects of forced *her5* expression on *her11* expression. Regarding the second possibility, identifying the CRE for *her11* will be necessary. Further experiments should introduce mutations at different positions in the *her5* gene to assess their effect on *her11* expression.

In *her3* mutants, *her3* expression was upregulated. This suggests the presence of a negative autoregulatory loop in *her3* regulation, although the underlying mechanism remains unknown. Notably, the expression of *her5*, *her6*, and *her9* was unaffected despite overlapping expression in the MHB (*her5*, *her9*) and IPCD‐r3 (*her6*), suggesting that *her3* does not regulate the expression of these three Notch‐independent *her* genes (Figure 1A).

Here, we also examined the expression of Notch‐independent *her* genes in recently established *her6* mutants (Tsuruoka et al. 2025). The expression of *her6* was decreased in *her6* homozygotes, raising the possibility of a positive feedback loop regulating *her6* expression. However, Her proteins are generally known as transcriptional repressors. Indeed, mouse Hes1, the ortholog of Her6, represses its own transcription, forming a negative regulatory loop (Kageyama et al. 2007). During NPC differentiation in the hindbrain, *her6* has been proposed to repress its own transcription in pharyngulae, leading to negative feedback (Soto et al. 2020), a hypothesis supported by mutant analysis at similar stages (Sigloch et al. 2023). One possible explanation for this discrepancy between previous studies and our current findings is that *her6* forms a positive feedback loop through an unknown repressor in the early neural plate. Alternatively, mutant *her6* mRNA may be degraded by mRNA quality control mechanisms such as nonsense‐mediated mRNA decay (NMD) (Neu‐Yilik et al. 2004). Nevertheless, the potential self‐regulation of *her6* requires further analysis.

The expression of *her3* and *her9*, which partially overlaps with *her6* expression (IPCD‐r3 and floor plate, respectively), remained unchanged in *her6* mutants. A previously reported weak upregulation of *her*9 in *her6* pharyngula mutants (Sigloch et al. 2023), which contrasts with our findings. It is likely that *her6* is not involved in the expression of *her3* and *her9*, at least at early somite stages.

Overall, although mutual regulation or self‐regulation was suggested in some contexts, the expression of Notch‐independent *her* genes appears to be generally independent. At least in terms of neurogenesis patterning in the neural plate, their mutations exhibited recessive inheritance. Our KO studies further showed that homozygotes for *her3*, *her5*, and *her11* developed normally to become fertile adults. Indeed, *neurog1* expression was restored at later stages in *her5* mutants. A similar observation was reported in KD experiments, though the possibility remained that KD effects gradually diminished over time. Our findings using *her5* mutants strongly corroborated the recovery of *neurog1* expression. The loss of a given *her* gene does not affect the expression of other *her* genes in most cases, suggesting that the generally limited developmental impact of *her* mutations is unlikely to be due to transcriptional adaptation, which could weaken mutation effects through the upregulation of related genes (Sztal and Stainier 2020); however, this possibility should be further investigated. Although each *her* gene has a unique expression pattern, its expression changes dynamically, which may facilitate compensatory mechanisms in mutants at later stages. Of note, the previously reported null mutation of *her3* did not cause lethality, similar to our mutants, although adult fish were smaller compared to WT fish (Kent et al. 2023). In contrast, *her9* mutants showed severe eye defects and were larval lethal (Coomer et al. 2020). Therefore, the later phenotypes of Notch‐independent *her* mutants likely reflect their distinct roles in subsequent development beyond early neurogenesis.

### 4.3 Collaboration Among Notch‐Independent *her* Genes in Neurogenesis Patterning

The expression patterns of Notch‐independent *her* genes are distinct yet partially overlapping, collectively shaping the characteristic patterns of NPPs and PCDs (Figure 1A). This overlap is expected to mask phenotypes in mutants of individual Notch‐independent *her* genes, where coexpressed *her* genes may compensate for their loss. Indeed, KD of individual *her* genes resulted in ectopic neurogenesis, but only in restricted regions rather than across their entire expression domains.

Interaction between *her5* and *her11* in regulating neurogenesis was previously examined (Ninkovic et al. 2005), suggesting that Her11 and Her5 are functionally redundant and that their combined activity inhibits neurogenesis in a dose‐dependent manner, with varying sensitivity thresholds along the medio‐lateral axis of the neural plate, thereby maintaining a NPP at the embryonic MHB (MIZ and LIZ). Similarly, combined KD of *her3* and *her9* resulted in much broader ectopic neurogenesis in the spinal cord compared to single KDs, further supporting the idea that the combined expression of these two *her* genes defines neurogenesis patterns (Bae et al. 2005). Additionally, Her8a has been suggested to maintain NPPs in r1/2 and r4 by forming heterodimers with Her3 (Webb et al. 2011). To the best of our knowledge, however, interactions between *her3* and *her6*, as well as *her3* and *her5*, have not yet been investigated.

In the current study, we examined *neurog1* expression in *her3/her6* double mutants. *her3* expression overlaps with the transverse stripe of *her6* exclusively in the IPCD of r3, where both stripes intersect, but there is no coexpression in other NPPs. In *her3/6* double homozygotes, the ectopic expression patterns of the two single mutants (*her3* mutants, IPCD‐r2 and IPCD‐r4; *her6* mutants, ANB, vrc/drc/vcc, r3, hindbrain medial axis) were simply additive, with no additional anomalies in the neurogenesis pattern. Thus, each *her* gene maintains NPPs within its own expression domain(s) independently of the other, with no significant genetic interaction. A similar conclusion was drawn for the interaction between *her3* and *her5* based on double mutant analyses. Taken together, Notch‐independent *her* genes function independently not only in their regulatory interactions but also in their roles in patterning the neural plate for primary neurogenesis.

### 4.4 Upstream Mechanisms Regulating Neurogenesis Patterns

The mechanisms underlying the formation of characteristic PCD patterns remain unclear. Recent studies have shown that *pou5f3*, the zebrafish orthologue of *Oct4*, exhibits an expression pattern similar to, but broader than, that of PCDs in the neural plage, suggesting a role for *pou5f3* in primary neurogenesis (Inomata et al. 2020). Indeed, functional suppression of Pou5f3 through dominant interference disrupted *her3* and *her5* expression in the neural plate during late gastrulation. Furthermore, microarray analyses suggest that this Class V POU gene is required for the expression of *her3* and *her5* at the same stage (Ikeda et al. 2025). The strong dependence of *her3* on *pou5f3* was also demonstrated in early embryos (up to the mid‐gastrula stage) (Onichtchouk et al. 2010). In this study, we observed that *pou5f3* expression in the hindbrain remained unchanged in the absence of Notch‐independent *her* genes, suggesting that Pou5f3 functions as an upstream regulator of at least some Notch‐independent *her* genes in the neural plate around the bud stage.

Our luciferase reporter assays in vitro demonstrated that *sox3* activates *her3*, suggesting that SoxB1 TFs, which are widely expressed in the neural plate, serve as additional activators for Notch‐independent *her* genes (Ikeda et al. 2025). Consistent with this, *her8a* expression has been shown to depend on SoxB (Webb et al. 2011). In contrast, the expression of Notch‐independent *her* genes, such as *her3* and *her9*, has been reported to be independent of proneural genes (Bae et al. 2005). In addition to positive regulation, other factors likely contribute to the establishment of NPD/PCD patterns, including repressive TFs and secreted signaling molecules. Various MHB‐ and rhombomere‐specific TFs, as well as FGFs, BMPs, and retinoic acid, have been implicated as upstream regulators of these processes (Bae et al. 2005; Moens and Prince 2002). However, further studies are required to reveal the precise mechanisms governing the patterning of primary neurogenesis.

## 5 Conclusion

This study investigated Notch‐independent *her* genes to elucidate the mechanisms underlying the patterning of neurogenesis in the early neural plate. By generating mutants for *her3, her5*, and *her11*, we confirmed their roles as pre‐pattern genes in the neural plate, as previously suggested by KD experiments. Our mutant analyses further demonstrated that Notch‐independent *her* genes function independently in both their regulatory interactions and their roles in neurogenesis patterning within the neural plate.

## Author Contributions

**Takero Ohyanagi:** conceptualization, data curation, formal analysis, investigation, methodology, visualization, writing – original draft. **Hiroki Tokizaki:** data curation, formal analysis, investigation, validation, visualization. **Takehisa Sato:** data curation, formal analysis, investigation, validation, visualization, writing – original draft. **Momo Tsuruoka:** data curation, formal analysis, investigation, visualization, writing – original draft. **Kyo Yamasu:** conceptualization, funding acquisition, project administration, resources; supervision, writing – review and editing.

## Ethics Statement

All experiments using live fish complied with the protocols approved by the Committee for Animal Care and Use of Saitama University.

## Supporting information


**Figure S1.** Expression of proneural cluster‐related genes in *her5* mutants. Offspring of heterozygotic crosses were stained by WISH at the bud stage, photographed, and then genotyped. Dorsal views of whole stained embryos are shown with anterior to the top. Enlarged views of the midbrain‐hindbrain regions are shown in Figure 8B. Arrows mark ectopic expression in MIZ. Numbers of the embryos with the indicated morphology and total scored embryos are shown in Figure 8. *her5* ^*+/−*^ embryos were indistinguishable from *her5* ^*+/+*^ embryos, and both were scored together and referred to as *her5* ^*+/*^. Images for *her5* ^*+/+*^ embryos are shown as representatives. See the legends to Figures 1 and 3 for abbreviations. Scale bar, 200 μm.

**Figure S2.** Expression of brain‐forming genes in *her5* mutants throughout early brain development. The expression of *neurog1* (A–D), *pax2a* (E, F), and *her11* (G–N) was examined at earlier and/or later stages than the bud stage. (A, B, E, F, K–N) Lateral views with anterior to the left and dorsal to the top. (C, D, G–J) Dorsal views with anterior to the left (C, D) or to the top (G–J). Four or more embryos for each gene and genotype were examined, showing the same expression patterns. *her5* ^*+/−*^ embryos were indistinguishable from *her5* ^*+/+*^ embryos, and both were scored together and referred to as *her5* ^*+/*^. Images for *her5* ^*+/+*^ embryos are shown as representatives. cg, cranial ganglia; os, optic stalk; pm, presomitic mesoderm; tec, tectum; teg, tegmentum. See the legends to Figures 1 and 3 for other abbreviations. Scale bars, 100 μm (A–F) or 200 μm (G–N).

Table S1.

## Data Availability

The data that support the findings of this study are available from the corresponding author upon reasonable request.

## References

[dvg70015-bib-0001] Alunni, A. , and L. Bally‐Cuif . 2016. “A Comparative View of Regenerative Neurogenesis in Vertebrates.” Development 143: 741–753. 26932669 10.1242/dev.122796 PMC4813331

[dvg70015-bib-0002] Archer, T. C. , J. Jin , and E. S. Casey . 2011. “Interaction of Sox1, Sox2, Sox3 and Oct4 During Primary Neurogenesis.” Developmental Biology 350: 429–440. 21147085 10.1016/j.ydbio.2010.12.013 PMC3033231

[dvg70015-bib-0003] Bae, Y.‐K. , T. Shimizu , and M. Hibi . 2005. “Patterning of Proneuronal and Inter‐Proneuronal Domains by Hairy‐ and Enhancer of Split‐Related Genes in Zebrafish Neuroectoderm.” Development 132: 1375–1385. 15716337 10.1242/dev.01710

[dvg70015-bib-0004] Baek, J. H. , J. Hatakeyama , S. Sakamoto , T. Ohtsuka , and R. Kageyama . 2006. “Persistent and High Levels of Hes1 Expression Regulate Boundary Formation in the Developing Central Nervous System.” Development 133: 2467–2476. 16728479 10.1242/dev.02403

[dvg70015-bib-0005] Beatus, P. , and U. Lendahl . 1998. “Notch and Neurogenesis.” Journal of Neuroscience Research 54: 125–136. 9788272 10.1002/(SICI)1097-4547(19981015)54:2<125::AID-JNR1>3.0.CO;2-G

[dvg70015-bib-0006] Belting, H.‐G. , G. Hauptmann , D. Meyer , et al. 2001. “ *Spiel Ohne Grenzen/pou2* Is Required During Establishment of the Zebrafish Midbrain‐Hindbrain Boundary Organizer.” Development 128: 4165–4176. 11684654 10.1242/dev.128.21.4165 PMC4027960

[dvg70015-bib-0007] Burgess, S. , G. Reim , W. Chen , N. Hopkins , and M. Brand . 2002. “The Zebrafish *Spiel‐Ohne‐Grenzen* (*Spg*) Gene Encodes the POU Domain Protein Pou2 Related to Mammalian *Oct4* and is Essential for Formation of the Midbrain and Hindbrain, and for Pre‐Gastrula Morphogenesis.” Development 129: 905–916. 11861474 10.1242/dev.129.4.905

[dvg70015-bib-0008] Caron, A. , L. Trzuskot , and B. W. Lindsey . 2022. “Uncovering the Spectrum of Adult Zebrafish Neural Stem Cell Cycle Regulators.” Frontiers in Cell and Development Biology 10: 941893. 10.3389/fcell.2022.941893 PMC9277145 35846369

[dvg70015-bib-0009] Chapouton, P. , K. J. Webb , C. Stigloher , et al. 2011. “Expression of Hairy/Enhancer of Split Genes in Neural Progenitors and Neurogenesis Domains of the Adult Zebrafish Brain.” Journal of Comparative Neurology 519: 1748–1769. 21452233 10.1002/cne.22599

[dvg70015-bib-0010] Chitnis, A. , D. Henrique , J. Lewis , D. Ish‐Horowicz , and C. Kintner . 1995. “Primary Neurogenesis in *Xenopus* Embryos Regulated by a Homologue of the *Drosophila* Neurogenic Gene *Delta* .” Nature 375: 761–766. 7596407 10.1038/375761a0

[dvg70015-bib-0011] Chitnis, A. B. , and I. B. Dawid . 1999. “Neurogenesis in Zebrafish Embryos.” Methods in Cell Biology 59: 367–386. 9891370 10.1016/s0091-679x(08)61835-x

[dvg70015-bib-0012] Coomer, C. E. , S. G. Wilson , K. F. Titialii‐Torres , et al. 2020. “Her9/Hes4 Is Required for Retinal Photoreceptor Development, Maintenance, and Survival.” Scientific Reports 10: 11316. 32647335 10.1038/s41598-020-68172-2 PMC7347560

[dvg70015-bib-0013] Corbin, J. G. , N. Gaiano , S. L. Juliano , S. Poluch , E. Stancik , and T. F. Haydar . 2008. “Regulation of Neural Progenitor Cell Development in the Nervous System.” Journal of Neurochemistry 106: 2272–2287. 18819190 10.1111/j.1471-4159.2008.05522.x PMC2640107

[dvg70015-bib-0014] Davis, R. L. , and D. L. Turner . 2001. “Vertebrate Hairy and Enhancer of Split Related Proteins: Transcriptional Repressors Regulating Cellular Differentiation and Embryonic Patterning.” Oncogene 20: 8342–8357. 11840327 10.1038/sj.onc.1205094

[dvg70015-bib-0015] del Diez Corral, R. , and K. G. Storey . 2001. “Markers in Vertebrate Neurogenesis.” Nature Reviews Neuroscience 2: 835–839. 11715060 10.1038/35097587

[dvg70015-bib-0016] Fehon, R. G. , P. J. Kooh , I. Rebay , et al. 1990. “Molecular Interactions Between the Protein Products of the Neurogenic Loci *Notch* and *Delta*, Two EGF‐Homologous Genes in Drosophila.” Cell 61: 523–534. 2185893 10.1016/0092-8674(90)90534-l

[dvg70015-bib-0017] Geling, A. , M. Itoh , A. Tallafuß , et al. 2003. “bHLH Transcription Factor Her5 Links Patterning to Regional Inhibition of Neurogenesis at the Midbrain‐Hindbrain Boundary.” Development 130: 1591–1604. 12620984 10.1242/dev.00375

[dvg70015-bib-0018] Geling, A. , C. Plessy , S. Rastegar , U. Strähle , and L. Bally‐Cuif . 2004. “Her5 Acts as a Prepattern Factor That Blocks *neurogenin1* and *coe2* Expression Upstream of Notch to Inhibit Neurogenesis at the Midbrain‐Hindbrain Boundary.” Development 131: 1993–2006. 15056616 10.1242/dev.01093

[dvg70015-bib-0019] Hans, S. , N. Scheer , I. Riedl , E. v. Weizsäcker , P. Blader , and J. A. Campos‐Ortega . 2004. “ *her3*, a Zebrafish Member of the *Hairy‐E(Spl)* Family, Is Repressed by Notch Signalling.” Development (Cambridge, England) 131, no. 12: 2957–2969. 10.1242/dev.01167. 15169758

[dvg70015-bib-0020] Hauptmann, G. , H.‐G. Belting , U. Wolke , et al. 2002. “ *Spiel Ohne Grenzen/pou2* Is Required for Zebrafish Hindbrain Segmentation.” Development 129: 1645–1655. 11923201 10.1242/dev.129.7.1645

[dvg70015-bib-0021] Hidalgo‐Sánchez, M. , A. Andreu‐Cervera , S. Villa‐Carballar , and D. Echevarria . 2022. “An Update on the Molecular Mechanism of the Vertebrate Isthmic Organizer Development in the Context of the Neuromeric Model.” Frontiers in Neuroanatomy 16: 826976. 35401126 10.3389/fnana.2022.826976 PMC8987131

[dvg70015-bib-0022] Hirata, H. , K. Tomita , Y. Bessho , and R. Kageyama . 2001. “ *Hes1* and *Hes3* Regulate Maintenance of the Isthmic Organizer and Development of the Mid/Hindbrain.” EMBO Journal 20: 4454–4466. 11500373 10.1093/emboj/20.16.4454 PMC125583

[dvg70015-bib-0023] Houart, C. , L. Caneparo , C. Heisenberg , K. Barth , M. Take‐Uchi , and S. Wilson . 2002. “Establishment of the Telencephalon During Gastrulation by Local Antagonism of Wnt Signaling.” Neuron 35: 255–265. 12160744 10.1016/s0896-6273(02)00751-1

[dvg70015-bib-0070] Ikeda, M. K. , Y. Kobayashi , Y. Nakayama‐Sadakiyo , et al. 2025. “Transcriptome Analysis Suggested Striking Transition Around the End of Epiboly in the Gene Regulatory Network Downstream of the *Oct4*‐Type Pou Gene in Zebrafish Embryos.” Dev. Growth Differ. (in press). 10.1111/dgd.70012 PMC12199784 40490365

[dvg70015-bib-0024] Imayoshi, I. , M. Sakamoto , M. Yamaguchi , K. Mori , and R. Kageyama . 2010. “Essential Roles of Notch Signaling in Maintenance of Neural Stem Cells in Developing and Adult Brains.” Journal of Neuroscience 30: 3489–3498. 20203209 10.1523/JNEUROSCI.4987-09.2010 PMC6634119

[dvg70015-bib-0025] Inomata, C. , T. Yuikawa , Y. Nakayama‐Sadakiyo , et al. 2020. “Involvement of an *Oct4*‐Related *PouV* Gene, *pou5f3/pou2*, in Neurogenesis in the Early Neural Plate of Zebrafish Embryos.” Developmental Biology 457: 30–42. 31520602 10.1016/j.ydbio.2019.09.002

[dvg70015-bib-0026] Kageyama, R. , T. Ohtsuka , and T. Kobayashi . 2007. “The Hes Gene Family: Repressors and Oscillators That Orchestrate Embryogenesis.” Development 134: 1243–1251. 17329370 10.1242/dev.000786

[dvg70015-bib-0027] Kageyama, R. , T. Ohtsuka , H. Shimojo , and I. Imayoshi . 2008. “Dynamic Notch Signaling in Neural Progenitor Cells and a Revised View of Lateral Inhibition.” Nature Neuroscience 11: 1247–1251. 18956012 10.1038/nn.2208

[dvg70015-bib-0028] Kageyama, R. , H. Shimojo , and T. Ohtsuka . 2019. “Dynamic Control of Neural Stem Cells by bHLH Factors.” Neuroscience Research 138: 12–18. 30227160 10.1016/j.neures.2018.09.005

[dvg70015-bib-0029] Katoh, M. , and M. Katoh . 2007. “Integrative Genomic Analyses on *HES/HEY* Family: Notch‐Independent *HES1*, *HES3* Transcription in Undifferentiated ES Cells, and Notch‐Dependent *HES1, HES5, HEY1, HEY2, HEYL* Transcription in Fetal Tissues, Adult Tissues, or Cancer.” International Journal of Oncology 31: 461–466. 17611704

[dvg70015-bib-0030] Kent, M. R. , D. Calderon , K. M. Silvius , et al. 2023. “Zebrafish her3 Knockout Impacts Developmental and Cancer‐Related Gene Signatures.” Developmental Biology 496: 1–14. 36696714 10.1016/j.ydbio.2023.01.003 PMC10054701

[dvg70015-bib-0031] Kikuta, H. , M. Kanai , Y. Ito , and K. Yamasu . 2003. “ *gbx2* Homeobox Gene Is Required for the Maintenance of the Isthmic Region in the Zebrafish Embryonic Brain.” Developmental Dynamics 228: 433–450. 14579382 10.1002/dvdy.10409

[dvg70015-bib-0032] Kimmel, C. B. , W. W. Ballard , S. R. Kimmel , B. Ullmann , and T. F. Schilling . 1995. “Stages of Embryonic Development of the Zebrafish.” Developmental Dynamics 203: 253–310. 8589427 10.1002/aja.1002030302

[dvg70015-bib-0033] Lamb, T. M. , and R. M. Harland . 1995. “Fibroblast Growth Factor is a Direct Neural Inducer, Which Combined With Noggin Generates Anterior‐Posterior Neural Pattern.” Development 121: 3627–3636. 8582276 10.1242/dev.121.11.3627

[dvg70015-bib-0034] Latimer, A. J. , J. Shin , and B. Appel . 2005. “ *her9* Promotes Floor Plate Development in Zebrafish.” Developmental Dynamics 232, no. 4: 1098–1104. 10.1002/dvdy.20264. 15739223

[dvg70015-bib-0035] Matsuda, M. , M. Koga , K. Woltjen , E. Nishida , and M. Ebisuya . 2015. “Synthetic Lateral Inhibition Governs Cell‐Type Bifurcation With Robust Ratios.” Nature Communications 6: 6195. 10.1038/ncomms7195 25652697

[dvg70015-bib-0036] Moens, C. B. , and V. E. Prince . 2002. “Constructing the Hindbrain: Insights From the Zebrafish.” Developmental Dynamics 224: 1–17. 11984869 10.1002/dvdy.10086

[dvg70015-bib-0037] Müller, M. , E. v. Weizsäcker , and J. A. Campos‐Ortega . 1996. “Transcription of a Zebrafish Gene of the *Hairy‐Enhancer of Split* Family Delineates the Midbrain Anlage in the Neural Plate.” Development Genes and Evolution 206, no. 2: 153–160. 10.1007/s004270050041. 24173468

[dvg70015-bib-0038] Mumm, J. S. , and R. Kopan . 2000. “Notch Signaling: From the Outside in.” Developmental Biology 228: 151–165. 11112321 10.1006/dbio.2000.9960

[dvg70015-bib-0039] Nakamura, H. 2001. “Regionalization of the Optic Tectum: Combinations of Gene Expression That Define the Tectum.” Trends in Neurosciences 24: 32–39. 11163885 10.1016/s0166-2236(00)01676-3

[dvg70015-bib-0040] Neu‐Yilik, G. , N. H. Gehring , M. W. Hentze , and A. E. Kulozik . 2004. “Nonsense‐Mediated mRNA Decay: From Vacuum Cleaner to Swiss Army Knife.” Genome Biology 5: 218. 15059251 10.1186/gb-2004-5-4-218 PMC395777

[dvg70015-bib-0041] Ninkovic, J. , A. Tallafuss , C. Leucht , et al. 2005. “Inhibition of Neurogenesis at the Zebrafish Midbrain‐Hindbrain Boundary by the Combined and Dose‐Dependent Activity of a New *Hairy/E(Spl)* Gene Pair.” Development 132: 75–88. 15590746 10.1242/dev.01525

[dvg70015-bib-0042] Onichtchouk, D. , F. Geier , B. Polok , et al. 2010. “Zebrafish Pou5f1‐Dependent Transcriptional Networks in Temporal Control of Early Development.” Molecular Systems Biology 6: 354. 20212526 10.1038/msb.2010.9 PMC2858445

[dvg70015-bib-0043] Ota, S. , Y. Hisano , M. Muraki , et al. 2013. “Efficient Identification of TALEN‐Mediated Genome Modifications Using Heteroduplex Mobility Assays.” Genes to Cells 18: 450–458. 23573916 10.1111/gtc.12050 PMC4834911

[dvg70015-bib-0044] Pasini, A. , D. Henrique , and D. G. Wilkinson . 2001. “The Zebrafish Hairy/Enhancer‐of‐Split‐Related Gene her6 Is Segmentally Expressed During the Early Development of Hindbrain and Somites.” Mechanisms of Development 100: 317–321. 11165489 10.1016/s0925-4773(00)00538-4

[dvg70015-bib-0045] Pauli, A. , T. G. Montague , K. A. Lennox , M. A. Behlke , and A. F. Schier . 2015. “Antisense Oligonucleotide‐Mediated Transcript Knockdown in Zebrafish.” PLoS One 10: e0139504. 26436892 10.1371/journal.pone.0139504 PMC4593562

[dvg70015-bib-0046] Rentzsch, F. , J. Bakkers , C. Kramer , and M. Hammerschmidt . 2004. “Fgf Signaling Induces Posterior Neuroectoderm Independently of BMP Signaling Inhibition.” Developmental Dynamics 231: 750–757. 15532058 10.1002/dvdy.20244

[dvg70015-bib-0047] Rex, M. , A. Orme , D. Uwanogho , et al. 1997. “Dynamic Expression of Chicken Sox2 and Sox3 Genes in Ectoderm Induced to Form Neural Tissue.” Developmental Dynamics 209: 323–332. 9215646 10.1002/(SICI)1097-0177(199707)209:3<323::AID-AJA7>3.0.CO;2-K

[dvg70015-bib-0048] Rhinn, M. , and M. Brand . 2001. “The Midbrain‐Hindbrain Boundary Organizer.” Current Opinion in Neurobiology 11: 34–42. 11179870 10.1016/s0959-4388(00)00171-9

[dvg70015-bib-0049] Rogers, C. D. , S. A. Moody , and E. S. Casey . 2009. “Neural Induction and Factors That Stabilize a Neural Fate.” Birth Defects Research. Part C, Embryo Today 87: 249–262. 19750523 10.1002/bdrc.20157 PMC2756055

[dvg70015-bib-0050] Ross, L. S. , T. Parrett , and S. S. Easter Jr. 1992. “Axonogenesis and Morphogenesis in the Embryonic Zebrafish Brain.” Journal of Neuroscience 12: 467–482. 1371313 10.1523/JNEUROSCI.12-02-00467.1992 PMC6575612

[dvg70015-bib-0051] Rothenaigner, I. , M. Krecsmarik , J. A. Hayes , et al. 2011. “Clonal Analysis by Distinct Viral Vectors Identifies Bona Fide Neural Stem Cells in the Adult Zebrafish Telencephalon and Characterizes Their Division Properties and Fate.” Development 138: 1459–1469. 21367818 10.1242/dev.058156

[dvg70015-bib-0052] Rubenstein, J. L. , K. Shimamura , S. Martinez , and L. Puelles . 1998. “Regionalization of the Prosencephalic Neural Plate.” Annual Review of Neuroscience 21: 445–477. 10.1146/annurev.neuro.21.1.445 9530503

[dvg70015-bib-0053] Sakuma, T. , S. Hosoi , K. Woltjen , et al. 2013. “Efficient TALEN Construction and Evaluation Methods for Human Cell and Animal Applications.” Genes to Cells 18: 315–326. 23388034 10.1111/gtc.12037

[dvg70015-bib-0054] Schier, A. F. , and W. S. Talbot . 1998. “The Zebrafish Organizer.” Current Opinion in Genetics and Development 8: 464–471. 9729724 10.1016/s0959-437x(98)80119-6

[dvg70015-bib-0055] Schmidt, R. , U. Strähle , and S. Scholpp . 2013. “Neurogenesis in Zebrafish ‐ From Embryo to Adult.” Neural Development 8: 3. 23433260 10.1186/1749-8104-8-3 PMC3598338

[dvg70015-bib-0056] Schulte‐Merker, S. , and D. Y. Stainier . 2014. “Out With the Old, in With the New: Reassessing Morpholino Knockdowns in Light of Genome Editing Technology.” Development (Cambridge) 141: 3103–3104. 10.1242/dev.112003 25100652

[dvg70015-bib-0057] Shimojo, H. , T. Ohtsuka , and R. Kageyama . 2011. “Dynamic Expression of Notch Signaling Genes in Neural Stem/Progenitor Cells.” Frontiers in Neuroscience 5: 78. 21716644 10.3389/fnins.2011.00078 PMC3116140

[dvg70015-bib-0058] Sigloch, C. , D. Spitz , and W. Driever . 2023. “A Network of Notch‐Dependent and ‐Independent Her Genes Controls Neural Stem and Progenitor Cells in the Zebrafish Thalamic Proliferation Zone.” Development 150: dev201301. 37009986 10.1242/dev.201301 PMC10112928

[dvg70015-bib-0059] Soto, X. , V. Biga , J. Kursawe , et al. 2020. “Dynamic Properties of Noise and Her6 Levels Are Optimized by miR‐9, Allowing the Decoding of the Her6 Oscillator.” EMBO Journal 39: e103558. 32395844 10.15252/embj.2019103558 PMC7298297

[dvg70015-bib-0060] Stigloher, C. , P. Chapouton , B. Adolf , and L. Bally‐Cuif . 2008. “Identification of Neural Progenitor Pools by E(Spl) Factors in the Embryonic and Adult Brain.” Brain Research Bulletin 75: 266–273. 18331883 10.1016/j.brainresbull.2007.10.032

[dvg70015-bib-0061] Sztal, T. E. , and D. Y. R. Stainier . 2020. “Transcriptional Adaptation: A Mechanism Underlying Genetic Robustness.” Development 147: dev186452. 32816903 10.1242/dev.186452

[dvg70015-bib-0062] Takke, C. , P. Dornseifer , E. v. Weizsacker , and J. A. Campos‐Ortega . 1999. “ *her4*, a Zebrafish Homologue of the *Drosophila* Neurogenic Gene *E(Spl)*, is a Target of NOTCH Signalling.” Development 126, no. 9: 1811–1821. 10.1242/dev.126.9.1811. 10101116

[dvg70015-bib-0063] Tallafuß, A. , and L. Bally‐Cuif . 2003. “Tracing of *her5* Progeny in Zebrafish Transgenics Reveals the Dynamics of Midbrain‐Hindbrain Neurogenesis and Maintenance.” Development 130: 4307–4323. 12900448 10.1242/dev.00662

[dvg70015-bib-0064] Tsuruoka, M. , H. Tokizaki , and K. Yamasu . 2025. Definition of the Characteristic Neurogenesis Pattern in the Neural Plate by the *Hes1* Orthologue Gene, *her6*, During Early Zebrafish Development. Cells and Development (in press). 10.1016/j.cdev.2025.20402. SSRN Preprint. https://ssrn.com/abstract=5092554. 40228713

[dvg70015-bib-0065] Umeda, K. , K. Tanaka , G. Chowdhury , K. Nasu , Y. Kuroyanagi , and K. Yamasu . 2024. “Evolutionarily Conserved Roles of *foxg1a* in the Developing Subpallium of Zebrafish Embryos.” Development, Growth & Differentiation 66: 219–234. 10.1111/dgd.12917 PMC11457518 38378191

[dvg70015-bib-0066] Webb, K. J. , M. Coolen , C. J. Gloeckner , et al. 2011. “The Enhancer of Split Transcription Factor Her8a is a Novel Dimerisation Partner for Her3 That Controls Anterior Hindbrain Neurogenesis in Zebrafish.” BMC Developmental Biology 11: 27. 21586122 10.1186/1471-213X-11-27 PMC3125270

[dvg70015-bib-0067] Weinmaster, G. 1997. “The Ins and Outs of Notch Signaling.” Molecular and Cellular Neurosciences 9: 91–102. 9245493 10.1006/mcne.1997.0612

[dvg70015-bib-0068] Wood, H. B. , and V. Episkopou . 1999. “Comparative Expression of the Mouse *Sox1*, *Sox2* and *Sox3* Genes From Pre‐Gastrulation to Early Somite Stages.” Mechanisms of Development 86: 197–201. 10446282 10.1016/s0925-4773(99)00116-1

[dvg70015-bib-0069] Yuikawa, T. , T. Sato , M. Ikeda , et al. 2024. “Elongation of the Developing Spinal Cord Is Driven by Oct4‐Type Transcription Factor‐Mediated Regulation of Retinoic Acid Signaling in Zebrafish Embryos.” Developmental Dynamics 253: 404–422. 37850839 10.1002/dvdy.666

