# Supplementary figures and images for: Genetic Mechanism That Defines the Characteristic Neurogenesis Patterns in the Neural Plate Using Hes/her Genes During Early Vertebrate Development
Source: Genesis. 2025 Jun 2;63(3):e70015. doi: 10.1002/dvg.70015 (PMC12127900; doi:10.1002/dvg.70015)

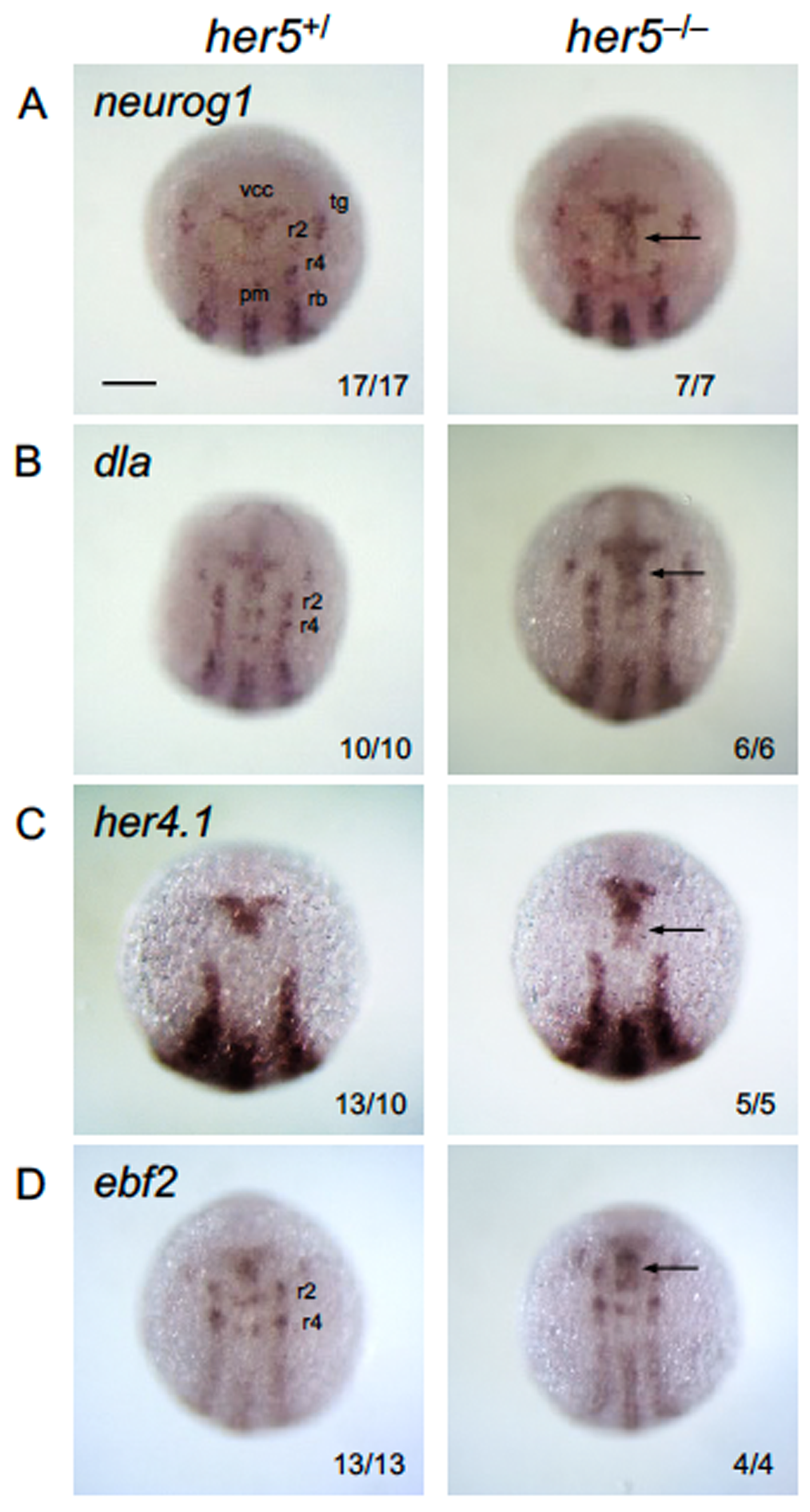

Supplement: Supplementary file 1 — Figure S1. Expression of proneural cluster‐related genes in her5 mutants. Offspring of heterozygotic crosses were stained by WISH at the bud stage, photographed, and then genotyped. Dorsal views of whole stained embryos are shown with anterior to the top. Enlarged views of the midbrain‐hindbrain regions are shown in Figure 8B. Arrows mark ectopic expression in MIZ. Numbers of the embryos with the indicated morphology and total scored embryos are shown in Figure 8. her5 +/− embryos were indistinguishable from her5 +/+ embryos, and both were scored together and referred to as her5 +/ . Images for her5 +/+ embryos are shown as representatives. See the legends to Figures 1 and 3 for abbreviations. Scale bar, 200 μm. [file DVG-63-e70015-s001.tif]

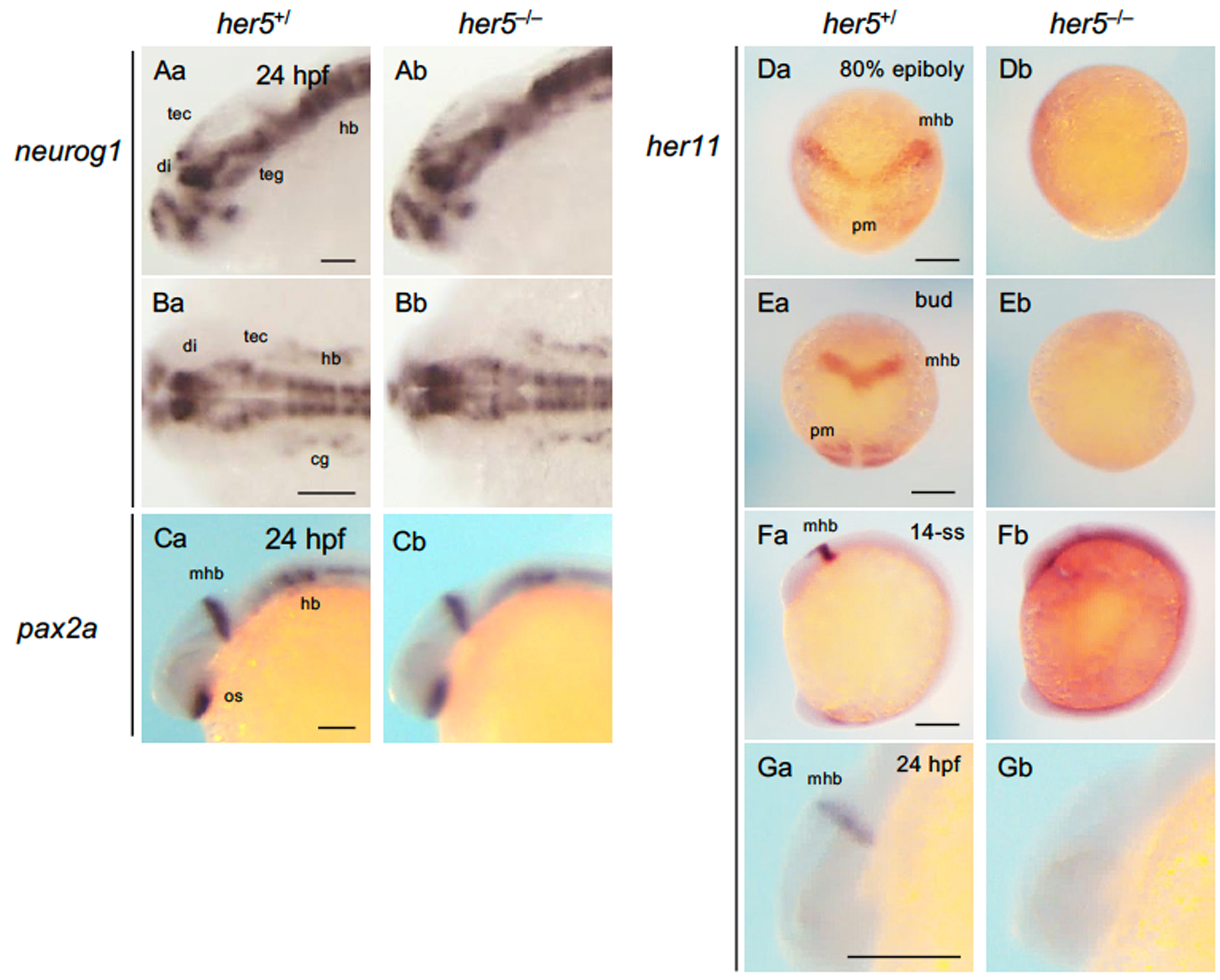

Supplement: Supplementary file 2 — Figure S2. Expression of brain‐forming genes in her5 mutants throughout early brain development. The expression of neurog1 (A–D), pax2a (E, F), and her11 (G–N) was examined at earlier and/or later stages than the bud stage. (A, B, E, F, K–N) Lateral views with anterior to the left and dorsal to the top. (C, D, G–J) Dorsal views with anterior to the left (C, D) or to the top (G–J). Four or more embryos for each gene and genotype were examined, showing the same expression patterns. her5 +/− embryos were indistinguishable from her5 +/+ embryos, and both were scored together and referred to as her5 +/ . Images for her5 +/+ embryos are shown as representatives. cg, cranial ganglia; os, optic stalk; pm, presomitic mesoderm; tec, tectum; teg, tegmentum. See the legends to Figures 1 and 3 for other abbreviations. Scale bars, 100 μm (A–F) or 200 μm (G–N). [file DVG-63-e70015-s003.tif]
